# Beyond Estrogenicity: A Comparative Assessment of Bisphenol A and Its Alternatives in In Vitro Assays Questions Safety of Replacements

**DOI:** 10.1021/acs.est.5c07018

**Published:** 2025-08-16

**Authors:** Vanessa Srebny, Luise Henneberger, Maria König, Julia Huchthausen, Jenny Braasch, Beate I. Escher

**Affiliations:** † Department of Cell Toxicology, 28342 Helmholtz Centre for Environmental Research − UFZ, Permoser Str. 15, Leipzig 04318, Germany; ‡ Environmental Toxicology, Department of Geosciences, Eberhard Karls University Tübingen, Schnarrenberger Str. 94-96, Tübingen 72076, Germany

**Keywords:** BPA, in vitro bioassay, new approach methodologies (NAM), bisphenol alternatives, metabolic activation

## Abstract

Bisphenol A (BPA) is a well-known endocrine disruptor linked to numerous adverse health outcomes and was, therefore, banned in food-contact materials in the European Union. Numerous alternatives are now in commerce, but their health hazards are often inadequately addressed. This study compared BPA and 26 alternatives in six in vitro bioassays for cytotoxicity, endocrine disruption, xenobiotic metabolism, adaptive stress responses, mitochondrial toxicity, and neurotoxicity. We developed a cumulative specificity ratio score that integrates the degree of specific activation and overall toxicological activity across a test battery, enabling direct comparison of BPA with its alternatives. Several alternatives with close structural resemblance showed similar or stronger activation of the estrogen receptor α (ERα) than BPA. The lack of estrogenicity for several BPA alternatives, e.g., 4-(4-phenylmethoxyphenyl)­sulfonylphenol (BPS-MPE), was accompanied by a shift toward peroxisome proliferator-activated receptor γ (PPARγ) activation, a receptor that is not relevant for BPA itself. Some alternatives additionally inhibited mitochondrial functions and caused neurotoxicity. Simulated phase I metabolism reduced the cytotoxicity of all alternatives except for methyl bis­(4-hydroxyphenyl)­acetate (Bz) and 4-[[4-(allyloxy)­phenyl]­sulfonyl]­phenol (BPS-MAE), while estrogenic activity remained unchanged or decreased. This study demonstrates the utility of bioassays for rapid hazard assessment and comparative evaluation, suggesting that many BPA alternatives are regrettable substitutes, although 2,2,4,4-tetramethyl-1,3-cyclobutanediol (TMCD) is a potentially more benign alternative.

## Graphical abstract

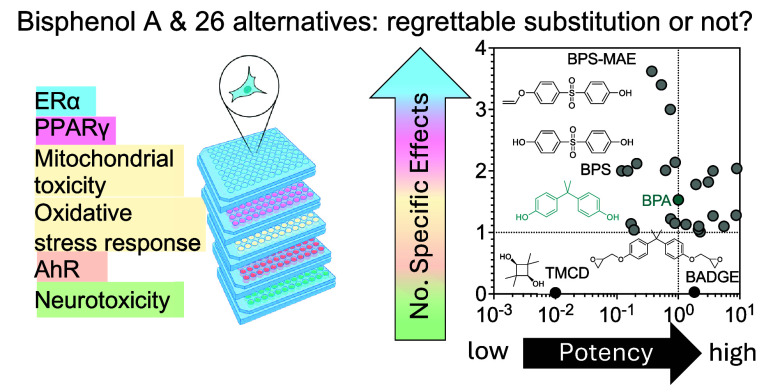

## 1 Introduction

Bisphenol A (BPA) has long been a key industrial chemical in the production of plastics and color developers, widely used in food packaging, dental fillings, and thermal paper.^1^ However, BPA is now widely recognized as a potent endocrine disruptor with established links to adverse human health effects^2,3^ and is considered a substance of very high concern (SVHC).^4^ Most recently, the European Food Safety Authority (EFSA) proposed a drastic reduction in the tolerable daily intake (TDI) from 4 μg/kg/day to 0.2 ng/kg/day,^5^ and the European Commission adopted a ban on BPA in food contact materials in December 2024 (Regulation 2024/3190).^6,7^

Previous regulatory restrictions and public awareness have driven the development of numerous BPA alternatives, with Bisphenol S (BPS) and Bisphenol F (BPF) representing the most widely used alternatives.^8^ Today, BPA alternatives are increasingly detected in the environment^9^ and humans,^10^ including the human placenta and cord blood.^11^ Given their structural resemblance to BPA, the question arises whether they are truly safe­(r) alternatives or if they present similar or even greater risks.^12^

Indeed, several alternatives, such as BPS,^13^ Bisphenol AF (BPAF),^14^ and 4,4′-(1,3-Dimethylbutylidene)­diphenol (BP-MIBK),^15^ have also been classified as reproductive toxicants under the European Union’s chemicals regulation REACH. Together with BPA, Bisphenol B (BPB) and Tetrabromobisphenol A (TBBPA) are also categorized as SVHCs.^4^ While these cases represent well-studied examples, toxicological data remain scarce for many other BPA alternatives.^16^

New Approach Methodologies (NAMs), particularly high-throughput cell-based in vitro assays, offer a promising strategy to close toxicological data gaps and accelerate the identification of potentially hazardous substitute chemicals without relying on animal data.^17,18^ NAMs rely on established adverse outcome pathways (AOPs) and quantify the easily accessible molecular initiating events (MIEs) or key events (KEs).^19,20^ Taking endocrine disruption as an example, activation of the estrogen receptor in reporter gene assays by BPA and other xenoestrogens has been shown to correlate well with the occurrence of in vivo estrogenic adverse outcomes.^21,22^

There already exists abundant in vitro estrogenicity and antiandrogenicity data for BPA and its alternatives,^16,23–26^ but comprehensive data sets on additional potentially relevant endpoints remain scarce. To address these limitations and close toxicological data gaps, this study investigates 25 BPA alternatives prioritized within the European Partnership for the Assessment of Risks from Chemicals (PARC) (Figure 1, Table 1, Figure S1, Table S1).^27^ We additionally included Tetramethyl Bisphenol F (TMBPF), a compound developed as a nonestrogenic alternative for use in can coatings.^28,29^

**Figure 1:**
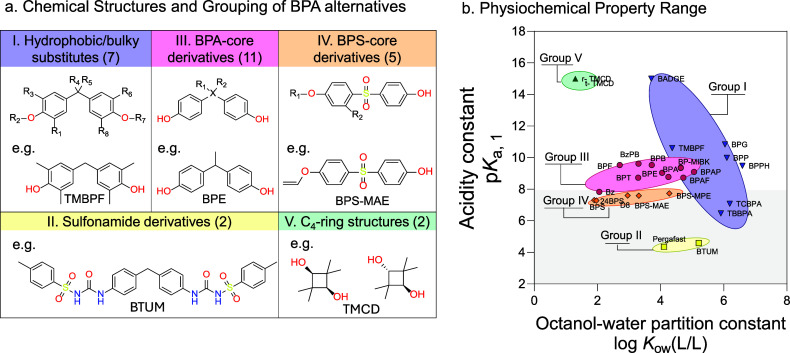
(a) Chemical structures and hierarchical clustering of the molecular fingerprints of the 26 bisphenol A (BPA) alternatives. Group I: Hydrophobic/bulky substituents (blue), Group II: Sulfonyl-amide derivatives (yellow), Group III: BPA-core derivatives (red), Group IV: BPS-core derivatives (orange), and Group V: C_4_-ring structures (green). For readability, only abbreviations are shown in the figure. BPA and all tested BPA alternatives (*N* = 27) and their abbreviations are listed in Table S1, and a complete list of structures is given in Figure S1. Depicted as examples for each structural group are as follows: (I) Tetramethyl bisphenol F (TMBPF), (II) 4,4’-Bis­(*p*-tolylsulfonylureido)­diphenylmethane (BTUM), (III) 4,4′-Ethylidenbisphenol (BPE), (IV) 4-[[4-(Allyloxy)­phenyl]­sulfonyl]­phenol (BPS-MAE), and (V) 2,2,4,4-Tetramethyl-1,3-cyclobutanediol (TMCD). (b) Relationship between the octanol–water partition constant (log *K*_ow_) and the acidity constants (p*K*_a_) of BPA alternatives. Physicochemical descriptors are provided in Table S1. Method of cluster analysis is described in Section 2.5.1, and results are presented in Section 3.1 and Text S4.

**Table 1:** Summary Table of Physicochemical Properties of Tested Bisphenol A Alternatives (Fraction of Neutral Species α_neutral_, Octanol–Water Partition Constant *K*_ow_, Ionization-Corrected Distribution Ratios between Liposomes and Water *D*_lipw_), Cytotoxicity IC_10_, Effect Concentrations EC_10_ and Efficacy Emax% in the ERα-UAS-bla GripTite Assay, PPARγ-UAS-bla 293H Assay, and Cumulative SR-Score (Σ-SR-Score) (eq 10)­^a^

Compound	α_neutral_ %	log *K*_ow_	log *D*_lipw_	IC_10,Median_ (M)	EC_10, ERα_ (M)	EC_10, PPARγ_ (M)	EC_10, AhR_ (M)	EC_IR1.5, ARE_ (M)	EC_10, MMP_ (M)	EC_10, NOI_ (M)	Emax%_ERα_	Emax%_PPARγ_	Σ-SR-Score
TCBPA	29	6.36	6.10	7.84 × 10^–5^	3.44 × 10^–6^	155 × 10^–7^			1.48 × 10^–5^	3.22 × 10^–4^	33	32	1.83
TBBPA	5	5.91	5.24	9.28 × 10^–5^		8.48 × 10^–8^			2.83 × 10^–5^		0	52	1.22
TMBPF	95	4.37	4.53	1.27 × 10^–5^		3.29 × 10^–7^				4.28 × 10^–6^	0	18	1.10
BPPH	99	6.67	6.85	8.02 × 10^–6^		5.55 × 10^–7^	9.54 × 10^–6^		1.96 × 10^–6^		0	12	1.28
BPG	100	6.01	6.19	7.84 × 10^–6^	9.02 × 10^–7^	1.17 × 10^–7^			3.12 × 10^–6^		19	22	2.04
BADGE	100	3.62	3.78	3.85 × 10^–5^						3.81 × 10^–5^			0.03
BPP	100	6.09	6.27	1.91 × 10^–5^	4.78 × 10^–7^				1.78 × 10^–6^		13		2.00
Pergafast	0.089	4.11	3.27	1.91 × 10^–5^		6.71 × 10^–7^	2.29 × 10^–5^			1.25 × 10^–5^		16	1.99
BTUM	0.0007	4.82	3.99	3.10 × 10^–5^		2.56 × 10^–7^				6.48 × 10^–5^		47	1.02
BPT	96	3.29	3.42	9.51 × 10^–5^	7.27 × 10^–7^	9.61 × 10^–7^			1.83 × 10^–5^		80	20	3.00
BPF	99	2.71	2.85	4.09 × 10^–4^	9.95 × 10^–7^				1.65 × 10^–4^		60		1.14
BPZ	99	4.64	4.80	5.28 × 10^–5^	4.43 × 10^–8^		8.04 × 10^–5^		2.21 × 10^–5^	3.05 × 10^–5^	21		1.13
BzPB	99	3.30	3.45	1.32 × 10^–4^	3.02 × 10^–6^	2.04 × 10^–6^		1.94 × 10^–5^	2.44 × 10^–5^		53	16	3.40
Bz	71	2.06	2.07	3.73 × 10^–4^	1.06 × 10^–6^		6.20 × 10^–5^				32		1.04
BPAP	95	5.06	5.22	3.31 × 10^–5^	1.94 × 10^–7^					5.12 × 10^–5^	24		1.05
BPAF	96	4.72	4.87	2.28 × 10^–5^	2.44 × 10^–8^				5.32 × 10^–6^	1.62 × 10^–5^	40		1.82
BP-MIBK	100	4.47	4.63	3.26 × 10^–5^	1.43 × 10^–8^				1.14 × 10^–5^		30		1.10
BPB	99	3.72	3.87	3.62 × 10^–5^	1.35 × 10^–7^				1.64 × 10^–5^	2.80 × 10^–5^	24		1.78
BPA	100	4.25	3.65	7.00 × 10^–5^	3.70 × 10^–7^		2.64 × 10^–4^		2.80 × 10^–5^	5.08 × 10^–5^	38		1.54
BPE	98	4.05	4.20	7.91 × 10^–5^	4.01 × 10^–7^		6.19 × 10^–5^		3.21 × 10^–5^	1.14 × 10^–4^	52		1.17
BPS-MPE	68	4.28	4.30	1.12 × 10^–4^		2.35 × 10^–7^			5.59 × 10^–6^	2.47 × 10^–4^		25	2.01
BPS-MAE	80	2.85	2.91	1.90 × 10^–4^	1.41 × 10^–5^	5.57 × 10^–7^			7.85 × 10^–5^	2.52 × 10^–5^	28	22	3.59
D8	61	2.87	2.83	3.37 × 10^–4^	1.12 × 10^–5^			1.22 × 10^–3^	5.20 × 10^–5^	1.91 × 10^–4^	18		2.12
BPS	39	1.97	1.77	5.61 × 10^–4^	1.43 × 10^–6^				1.17 × 10^–4^		66		2.00
2,4-BPS	43	1.90	1.72	4.63 × 10^–4^	1.98 × 10^–5^				5.90 × 10^–5^		19		2.00
t-TMCD	100	1.30	1.43	8.94 × 10^–3^	3.03 × 10^–3^				1.85 × 10^–2^	4.15 × 10^–3^			0.02
r-TMCD	100	1.30	1.43	8.29 × 10^–3^	1.29 × 10^–2^					5.99 × 10^–3^			0.01

a Details are given in Tables S1, S5, and S6.

We compiled the in vitro test battery based on a previous reanalysis of ToxCast/Tox21 data^17^ and identified assays where several bisphenols showed high specificity ratios (SR_baseline_) (Table S2).^9^ As expected, this included estrogen receptor α (ERα) agonism and also peroxisome proliferator-activated receptor γ (PPARγ) agonism and mitochondrial membrane potential (MMP) inhibition. Additionally, we included assays for oxidative stress response (ARE), aryl hydrocarbon receptor (AhR) agonism, and neurotoxicity—less explored pathways for BPA alternatives.^30^

We hypothesized that BPA alternatives closely resembling the core structure of BPA, such as BPB, would exhibit similar toxicological profiles, while structurally dissimilar alternatives, such as 2,2,4,4-tetramethyl-1,3-cyclobutanediol (TMCD) and *N*-(*p*-toluenesulfonyl)-*N*′-(3-(*p*-toluenesulfonyloxy)­phenyl)­urea (Pergafast), could potentially trigger other mechanisms and exhibit dissimilar toxicological profiles. Using BPA as a reference, we quantified relative effect potencies (REP_BPA_) and developed a novel scoring system for specific activation (SR-Score) to characterize toxicological profiles and mechanistic promiscuity.

Another major challenge in in vitro toxicology remains the limited availability of toxicokinetic information,^31^ with few studies covering BPA alternatives.^32,33^ Therefore, we activated the BPA alternatives with an abiotic cytochrome P450 catalyst (aCYP) before the ERα bioassay to simulate Phase I metabolism.^34^ Oxidation by aCYP cannot simulate all in vivo biotransformation processes but can be used to reveal whether Phase I oxidation leads to the formation of more or less toxic metabolites.

The combination of high-throughput bioassays with a novel scoring system enabled the identification of BPA alternatives with similar or greater hazard potential compared with BPA, as well as compounds that may represent more benign substitutes. This case study provides many concepts and tools to support in vitro-based read-across, prioritization, and substitution strategies that may also be applied to other chemical groups beyond bisphenols.

## 2 Materials and Methods

### 2.1 Chemicals

Full chemical names, identifiers and abbreviations are given in Table S1 (Figure 1a and Figure S1 for structural information). The test chemicals were dissolved in methanol (LC-MS grade, Chemsolute, Th. Geyer, Renningen, Germany) as 10 g/L stock solutions, with the exception of 0.05 g/L 4,4′-bis­(*p*-tolylsulfonylureido)-diphenylmethane (BTUM) and 0.1 g/L Pergafast, which were dissolved in acetonitrile (UHPLC-MS grade, Chemsolute, Th. Geyer, Renningen, Germany). The biomimetic catalyst 5,10,15,20-tetrakis-(2,6-dichlorophenyl)-porphyrin manganese (III) chloride (TDCPP, CAS 91463-17-1) was obtained from abcr (Karlsruhe, Germany). Ammonium acetate was sourced from Honeywell (Charlotte, NC, USA), formic acid from Serva (Heidelberg, Germany), and hydrogen peroxide (30% aqueous solution) from Sigma-Aldrich (St. Louis, MO, USA) Catalase from *Micrococcus lysodeikticus* (catalog number 60634) was also obtained from Sigma-Aldrich, with a batch activity of 123 945 U/mL.

### 2.2 Physicochemical Properties

Physicochemical properties provide input parameters for the baseline toxicity model. Acidity constants (p*K*_a_) and octanol–water partition constants of the neutral species (*K*_ow_) were measured with a Sirius T instrument from Pion Inc. (Sussex, UK) (Text S1.2 and Table S1). A detailed description of the p*K*_a_ and *K*_ow_ measurements can be found in literature.^35,36^

If experimental data were not available, we relied on literature data and established models (Text S1.3 and Table S1) to derive the ionization-corrected distribution ratios between biomembranes (liposomes) and water *D*_lip/w_, between bovine serum albumin (BSA) and water *D*_BSA/w_, and between structural proteins and water *D*_SP/w_.^37^

### 2.3 Bioassays

All bioassay media components and the ERα-UAS-bla GripTite and PPARγ-UAS-bla cells were obtained from Thermo Fisher Scientific (Waltham, MA, USA). AREc32 reporter gene cells based on MCF7 were provided by C. Roland Wolf (Cancer Research UK); AhR CALUX cells (H4L7.5c2 derived from H4IIe) were provided by Michael Denison (UC Davis, USA); and SH-SY5Y cells were purchased from Sigma-Aldrich.

Cell culture conditions and bioassay protocols including response detection are provided in Text S2.1–2.4 and Table S3.^38–42^ Cell viability was determined from cell confluency using an IncuCyte S3 Live-Cell Analysis System (Sartorius AG, Essen, Germany) before and after exposure, except for SH-SY5Y cells (live–dead staining with Nuclear Green LcS1 and propidium iodide, Abcam, UK). For metabolic activation experiments, cell viability was assessed using the ToxBLAzer DualScreen Kit (Thermo Fisher Scientific) to capture the remaining toxicity in ERα-UAS-bla GripTite after treatment with the aCYP catalyst (details in Text S2.4).

### 2.4 Oxidation Reaction Test of BPA and Its Alternatives with Abiotic CYP Catalyst

The protocol for the abiotic CYP oxidation is detailed in Henneberger et al.^34^ The reaction mixture consisted of 0.12 mM TDCPP (abiotic CYP catalyst), 143 mM ammonium acetate, 143 mM H_2_O_2_, and a bisphenol stock solution at a concentration 11× higher than the dosing solution for the bioassays. The mixtures were adjusted to a final volume of 100 μL with acetonitrile (ACN). The reaction mix was incubated for 15 min at 30 °C and 1000 rpm on a Bioshake iQ orbital shaker from QInstruments (Jena, Germany). Additionally, a mixture without test compounds was prepared as a control. Subsequently, 10 μL of the reaction mixture was transferred into a conical dosing vial and evaporated under a nitrogen flow. 100 μL of reaction mix was added for Pergafast and BTUM due to their low solubility in ACN.

For bioassay testing, the residue was redissolved in 120 μL of assay medium, supplemented with 10 μL of catalase solution, and incubated for 10 min at 30 °C. For chemical analysis, 10 μL of the reaction mixture was concentrated under vacuum (CentriVap, Labconco, Kansas City, MO, USA), diluted to 1 mg/L in ACN or ethyl acetate. The chemical analysis of the reaction mixtures was performed using liquid chromatography (LC) and gas chromatography (GC) coupled with various detection techniques, preferably Multiple Reaction Monitoring (MRM). Data acquisition and processing were carried out using the software MassHunter. Quantification was based on external calibrations. A detailed summary of all analytical conditions is provided in Text S3 and Tables SB–E.

### 2.5 Modeling

#### 2.5.1 Chemical Fingerprint Similarity Clustering

The similarity analysis was conducted using ECFP4 Morgan fingerprints in R (version 4.4.2, RStudio release 2024.09.1), employing the rcdk fingerprint package.^43,44^ Chemical similarities were quantified by calculating a Tanimoto distance matrix, which served as the basis for hierarchical clustering of the BPA alternatives into five distinct groups (Text S4, Figures S3–5, Table SF).

#### 2.5.2 Baseline Toxicity

The nominal baseline toxicity concentration causing 10% cytotoxicity (IC_10,baseline_) can be predicted from the critical membrane concentration IC_10,membrane_ of 0.026 mol/L_membrane_, which is uniform across all chemicals and cell types (eq 1).^45^ Further details are provided in Text S1.3, with volume fraction (VF) of proteins and lipids given in .^37^ The IC_10,baseline_ is essential for calculating the Toxic Ratio (TR) and Specificity Ratio (SR_baseline_).

$${\mathrm{IC}}_{10,\mathrm{baseline}}\left(M\right)=\frac{\mathrm{0.026 M}}{D_{\mathrm{lip}/w}}\times ({1+D}_{\mathrm{BSA}/w}\times {\mathrm{VF}}_{\mathrm{protein},\mathrm{medium}}+D_{\mathrm{lip}/w}\times {\mathrm{VF}}_{\mathrm{lipid},\mathrm{medium}})$$ 1

### 2.6 Data Analysis

#### 2.6.1 Bioassay Data Evaluation

An R workflow AutomatedBioassayScreening (R1.3.1, R-Studio Version 2025.05.0) was used (https://git.ufz.de/braung/automatedbioassayscreening)^46^ to derive the inhibitory concentration causing 10% cytotoxicity (IC_10_) and the effective concentration resulting in 10% of the maximum effect of the reference chemical (EC_10_) as detailed in Text S2.5. For ARE, the concentration that triggered an induction ratio of 1.5 (EC_IR1.5_) was used as the benchmark value, as detailed in Text S2.5. IC_10_ values also served as a threshold for data analysis in all assays; only concentrations below IC_10_ were considered when deriving EC_10_ or EC_IR1.5_.

The maximum efficacy (Emax%) refers to the maximum of the log–logistic concentration–response curve attained by test chemicals compared to the reference chemical’s maximum and was obtained for both ERα and PPARγ.

#### 2.6.2 Relative Effect Potency (REP_BPA_)

The REP_BPA_ (eq 2) was calculated as the ratio of the EC_10_ of BPA to the EC_10_ of the test compound and was used to compare the potencies of BPA alternatives (BP_*x*_) to BPA.

2 $${\mathrm{REP}}_{\mathrm{BPA}}=\frac{{\mathrm{EC}}_{10,\mathrm{BPA}}}{{\mathrm{EC}}_{{10,\mathrm{BP}}_{x}}}$$

#### 2.6.3 Toxic Ratio (TR)

The TR was calculated as the ratio of the predicted baseline cytotoxicity (IC_10,baseline_) to the experimental inhibitory concentration at 10% response (IC_10_) as shown in eq 3.^47^ TR indicates whether the cytotoxicity is triggered by specific toxicity mechanisms (TR ≥ 10) or not (0.1< TR< 10).

3 $$\mathrm{TR}=\frac{{\mathrm{IC}}_{10,\mathrm{baseline}}}{{\mathrm{IC}}_{10}}$$

#### 2.6.4 Specificity Ratio (SR_cytotoxicity_ and SR_baseline_)

The SR_cytotoxicity_ (eq 4) indicates whether a compound’s effect occurs near cytotoxic concentrations (SR = 1). This usually leads to nonspecific upregulation of many cellular processes.^48–50^ If experimental IC_10_ values were not measurable, we derived SR_baseline_ instead as a fallback using the corresponding baseline toxicity concentration IC_10,baseline_ (eq 5).

4 $${\mathrm{SR}}_{\mathrm{cytotoxicity}}=\frac{{\mathrm{IC}}_{10}}{{\mathrm{EC}}_{10}\;{\mathrm{or}\;\mathrm{EC}}_{\mathrm{IR1.5}}}$$

5 $${\mathrm{SR}}_{\mathrm{baseline}}=\frac{{\mathrm{IC}}_{10,\mathrm{baseline}}}{{\mathrm{EC}}_{10}\;{\mathrm{or}\;\mathrm{EC}}_{\mathrm{IR1.5}}}$$

We also reanalyzed the Tox21 data of Adamovsky et al.^9^ with the updated baseline toxicity prediction model (Table S2).^45^

#### 2.6.5 Toxicokinetic Ratio (TK-Ratio)

The TK-Ratio method was used to evaluate the impact of abiotic CYP oxidation on the potency of tested compounds. It was calculated as the ratio of the inhibitory or effect concentration at 10% response before oxidation (IC_10, before oxidation_ or EC_10, before oxidation_) to the corresponding IC_10_ or EC_10_ after oxidation (IC_10, after oxidation_ or EC_10, after oxidation_) (eq 6 with its standard error of mean, SEM) (eq 7). A TK-Ratio of 1 corresponds to a reaction mix with similar potency to the parent molecule, while a TK-Ratio >1 suggests a mix with elevated potency after oxidation. Finally, TK-Ratio <1 indicates a loss of potency after oxidation.

6 $$\mathrm{TK‐Ratio}=\frac{{\mathrm{IC}}_{10,\mathrm{before oxidation}}}{{\mathrm{IC}}_{10,\mathrm{after oxidation}}},\mathrm{or},\frac{{\mathrm{EC}}_{10,\mathrm{before oxidation}}}{{\mathrm{EC}}_{10,\mathrm{after oxidation}}}$$

7 $$\mathrm{SEM}(\mathrm{TK‐Ratio})=\sqrt{{\left(\frac{{\mathrm{SE}}_{\mathrm{IC10},\mathrm{before oxidation}}}{{\mathrm{IC}}_{10,\mathrm{after oxidation}}}\right)}^{2}+{\left(\frac{{\mathrm{IC}}_{10,\mathrm{before oxidation}}\times {\mathrm{SE}}_{\mathrm{IC10},\mathrm{after oxidation}}}{({\mathrm{IC}}_{10,\mathrm{after oxidation}}{)}^{2}}\right)}^{2}}$$

#### 2.6.6 *C*_parent_-Ratio

The C_parent_-Ratio quantifies the extent of oxidation of the parent compound after the abiotic CYP reaction. It was calculated as the ratio of the parent compound concentration after oxidation (*C*_parent, after oxidation_) to its concentration before oxidation (*C*_parent, before oxidation_) as shown in eq 8. A *C*_parent_-Ratio of 1 indicates no oxidation of the parent compound, while *C*_parent_-Ratio <1 suggests partial degradation or other losses.

8 $$C_{\mathrm{parent}}‐\mathrm{Ratio}=\frac{C_{\mathrm{parent},\mathrm{after oxidation}}}{C_{\mathrm{parent},\mathrm{before oxidation}}}$$

#### 2.6.7 SR-Score and Cumulative SR-Score (Σ-SR-Score)

We introduce the Specificity Ratio Score (SR-Score, eq 9) that classifies whether an individual assay is specifically activated (positive hit) by applying a sigmoidal transformation to the SR (eq 4), which rescales it to a range between 0 and 1. An SR of 1 corresponds to an SR-Score of 0, SR of 5 will be transformed to an SR-score of 0.5 while SR ≥10 results in an SR-Score of 1.

9 $$\mathrm{SR‐Score}=\frac{1}{{1+e}^{-(\mathrm{SR‐5})}}$$

To capture the overall extent of specific activation in the test battery, we summed the SR-Scores across all active end points (Assay_*i*_), resulting in the Cumulative SR-Score (Σ-SR-Score) (eq 10). The maximum Cumulative SR-Score is predetermined by the number of assays in the test battery—in our case, six if all bioassays are specifically activated.

10 $$\Sigma \mathrm{‐SR‐Score}=\overset{n}{\underset{i}{\sum }}\left(\frac{1}{{1+e}^{{-(\mathrm{SR}}_{i}‐5)}}\right)$$

## 3 Results and Discussion

### 3.1 Similarity Grouping of BPA and Its Alternatives

The 26 BPA alternatives and BPA were grouped by clustering Morgan fingerprints for their chemical similarity, as detailed in Text S4, revealing five distinct groups (Figure 1). Group I comprises seven compounds that are *ortho*- and *para*-substituted and are among the most hydrophobic BPA alternatives, including 2,2-Bis (2-hydroxy-5-biphenylyl)­propane (BPPH), TBBPA, and TMBPF (Table 1). Group II consists of the two phenol-free alternatives, Pergafast and BTUM, which contain sulfonyl urea bonds and differ significantly from BPA in their structure. Group III encompasses 12 BPA alternatives that are characterized by their close similarity to BPA (e.g., BPB, BP-MIBK, BPAF), differing only by structures around the central carbon atom. Group IV contains five BPA alternatives with a sulfonyl group substituting the central carbon as the core structure, such as BPS itself, 4-[[4-(allyloxy)­phenyl]­sulfonyl]­phenol (BPS-MAE), 4-[4-(benzyloxy)­benzenesulfonyl]­phenol (BPS-MPE), and 4-((4-isopropoxyphenyl)­sulfonyl)­phenol (D8, BPSIP). Finally, TMCD in both *trans* and as racemate forms, constitutes a fifth group, representing a C_4_-ring compound that is the most structurally distinct molecule in the present study.

### 3.2 Physiochemical Properties

BPA and its alternatives display a wide range of physicochemical properties (Figure 1b and Table S1). *K*_ow_ spans six log units (Figure 1b and Table 1), with BPA in the middle (log *K*_ow_ = 4.25). TMCD, BPS, and 2,4-Bisphenol S (2,4-BPS) exhibit the lowest log *K*_ow_ of 1.3–2, while chemicals such as BPPH and 4,4’-[1,4-Phenylenebis­(1-methylethylidene)] bisphenol (BPP) are at the other end of the scale (log *K*_ow_ of 6-7). Most compounds are neutral at pH 7.4, but 10 compounds deprotonate to mono- or doubly charged species, with BTUM and Pergafast representing the most acidic compounds (Figure 1b).

Stability tests in phosphate-buffered saline (PBS, pH 7.4, 37 °C, 48 h) revealed that most bisphenol alternatives remained stable under these conditions (Text S1.4 and ) except for Bisphenol A diglycidyl ether (BADGE) and Pergafast, consistent with the literature.^51,52^ Thus, the observed effects for these compounds will also reflect their lower parent concentration and potential contributions from hydrolysis products.

### 3.3 Cytotoxicity

We measured the cytotoxicity of BPA and its alternatives in HEK293H (PPARγ), HEK293T (ERα), H4IIe (AhR CALUX), MCF7 (ARE/MMP), and differentiated SH-SY5Y cells (Table S6). Overall, cytotoxicity was largely consistent across cell types, with IC_10_ values of individual compounds typically varying less than 1 order of magnitude from the median (Figure S6a and Table 1).

When comparing the different BPA alternatives, IC_10_ values spanned 3 orders of magnitude (3 to 9000 μM; Figure S6b). Two TMCD compounds exhibited the lowest overall cytotoxicity (IC_10_ of 8.3–8.9 mM in H4IIe, >18 mM in other cell lines), while most of the other compounds clustered within a factor of 10 around BPA, except for the more cytotoxic BPPH and TMBPF.

Previous studies applying avian cell lines have also reported that most BPA alternatives exhibit similar cytotoxicity to BPA.^53–55^ The very high IC_10_ for the two TMCDs was likely due to their low hydrophobicity and molecular size, which lead to reduced membrane accumulation (Figure 1b and Table 1). The IC_10_ of TMCD was within a factor of 10 of the cytotoxicity previously reported by Son et al.,^56^ who observed statistically significant reduced cell viability at 0.35 mM in keratinocytes. In contrast, the most hydrophobic compounds, namely BPG and BPPH were also the most cytotoxic.

### 3.4 Toxic Ratio (TR)

The TR compares observed cytotoxicity with baseline toxicity expected from nonspecific interaction with the biological membrane. The diagonal lines in Figure 2, where IC_10,baseline_ is plotted against the experimental IC_10_ of all cell lines, represent TR values of 0.1, 1, and 10. The baseline toxicity range is 0.1 < TR < 10, whereas TR ≥10 suggest additional, specific mechanisms such as oxidative stress. None of the TR values of the test chemicals exceeded 10 (Table S6 and Figure 2), which indicates that the acute toxicity of BPA and its alternatives is largely driven by baseline toxicity. Unexpectedly, the reactive epoxide BADGE was classified as a baseline toxicant with TR values ranging from 0.32 to 0.61 (Table S6), presumably due to the instability of the epoxide ring and rapid hydrolysis, so that the hydrolysis product, which is more hydrophilic, explains the low TR.

**Figure 2:**
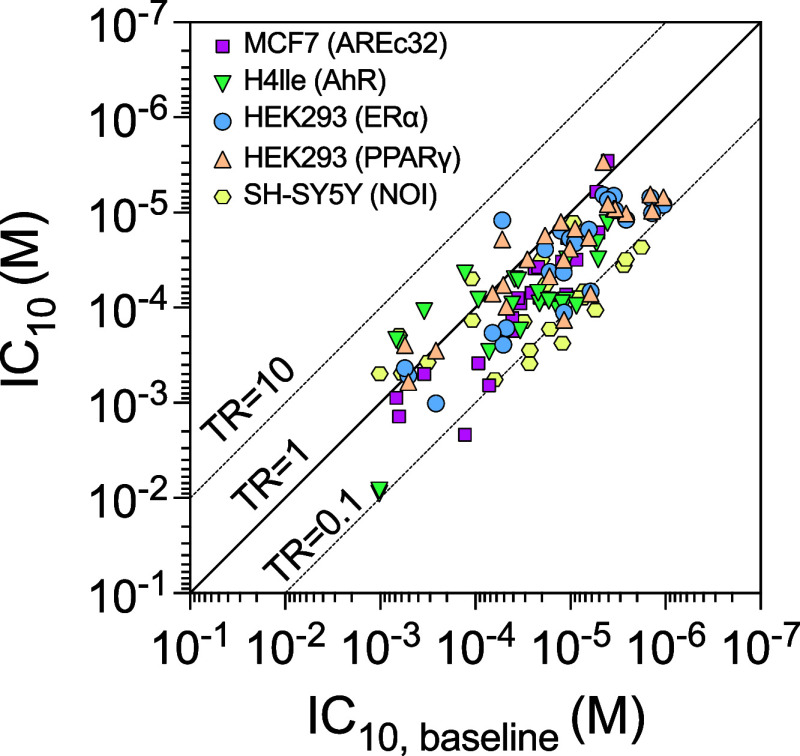
Toxic Ratio (TR) analysis compares experimental cytotoxicity IC_10_ (*y*-axis) with predicted baseline toxicity IC_10,baseline_ (*x*-axis) across different cell lines. Note that the axes are inversed because a lower IC_10_ corresponds to higher cytotoxicity. The diagonal line represents TR = 1, indicating no difference between baseline and experimental IC_10_ values, with dashed lines at TR = 0.1 and TR = 10 defining the baseline toxicity range. Symbols represent different cell lines: HEK293 (ERα), H4IIe (AhR), MCF7 (ARE), HEK293 (PPARγ), and SH-SY5Y. Data are provided in Table S6.

### 3.5 BPA and Its Alternatives Activate ERα, PPARγ, and Disturb Mitochondrial Membrane Potential

In this section, we describe the activity profiles of BPA and its alternatives for ERα, PPARγ, and AhR activation; decrease in the mitochondrial membrane potential (MMP); activation of the oxidative stress response (ARE); and neurite outgrowth inhibition (NOI), and compare their relative effect potencies (REP_BPA_), specificity ratios (SR), and specificity ratio scores to those of BPA.

#### 3.5.1 Estrogen Receptor α (ERα)

ERα agonism is the most studied and relevant end point for BPA and its alternatives,^16^ and here, 19 BPA alternatives activated ERα-UAS-bla GripTite above 10%. BPA itself (EC_10_ = 370 nM) was 18 700 times less potent than the reference estradiol (E2) (EC_10_ = 19.8 pM) and reached only 38% of the maximum effect of E2 (Table 1, Text S5.3 and ). Most BPA alternatives exhibited EC_10_ values within a factor of 10 compared to BPA (Table 1 and Figure 3) and were only partial agonists for ERα, with Emax% values ranging from 12 to 40% (Table 1). Prominent exceptions were 4,4’-thiodiphenol (BPT), achieving up to 80% of the maximum effect induced by E2, followed by BPS (66%), BPF (60%), and Bisphenol E (BPE, 52%) (Figure S8).

**Figure 3:**
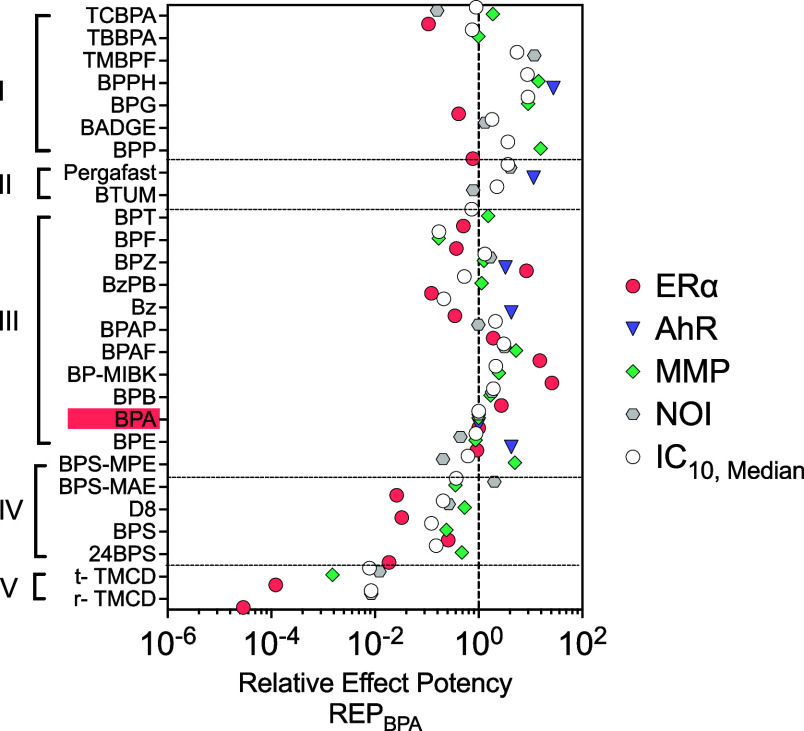
Relative effect potency in relation to BPA (REP_BPA_). Downward triangles represent AhR, red circles represent ERα, green diamonds represent mitochondrial membrane potential (MMP) inhibition, gray hexagons represent neurite outgrowth inhibition (NOI) in differentiated SH-SY5Y, and white circles represent median cytotoxicity IC_10,median_. Data are provided in Table S6. BPA and its alternatives are categorized into different groups (I–V) based on their structural similarity.

Particularly potent and specific alternatives hardly differed from BPA’s core structure, especially those in Group III. For instance, BP-MIBK has only an additional propyl group and was 26 times more potent than BPA (expressed as relative effect potency, REP_BPA_ = 26, Figure 3). Four other compounds exhibited higher potencies than BPA in our data set: BPAF (REP_BPA,ERα_ = 15), Bisphenol Z (BPZ), (REP_BPA,ERα_ = 8), BPB (REP_BPA,ERα_ = 3), and 4,4’-(1-phenylethylidene)­bisphenol (BPAP) (REP_BPA,ERα_ = 1.9). Among the least potent BPA alternatives were trans-TMCD (REP_BPA,ERα_ = 1.2 × 10^–4^) and racemic TMCD (REP_BPA,ERα_ = 2.9 × 10^–5^), as well as the BPS derivatives D8 (REP_BPA,Erα_ = 0.033) and BPS-MAE (REP_BPA,Erα_ = 0.026) and 2,4-BPS (REP_BPA,ERα_ = 0.019). Seven BPA alternatives did not activate ERα at noncytotoxic concentrations: TBBPA, TMBPF, BPPH, BADGE, Pergafast, BTUM and 2,4-BPS, BPS-MPE, (Table 1).

We observed high specific activation of ERα for BPA (SR_cytotoxicity_ = 115) and most of the 19 active BPA alternatives (Figure 4a, S7b, and Table S6), with particularly high SR for Bz (SR_cytotoxicity_ = 957), BP-MIBK (SR_cytotoxicity_ = 515), and BPAF (SR_cytotoxicity_ = 388) (Figure 4a).

**Figure 4:**
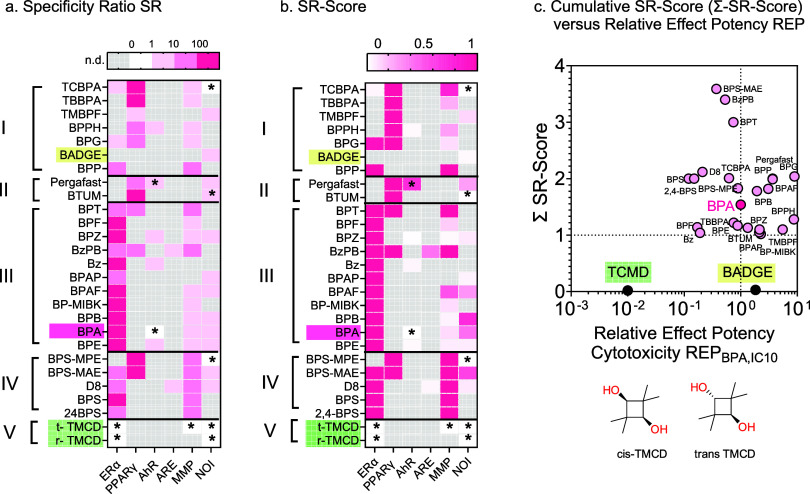
(a) Heatmap showing the Specificity Ratio (SR), defined as the ratio of experimental IC_10_ to EC_10_. Color coding: SR >100 (deep pink, high specificity), SR >10 (medium pink, medium specificity), 1< SR < 10 (light pink, moderate specificity), SR <1 (white, effects close to IC_10_). (b) Heatmap showing SR-Scores derived from the SR values. Deep pink tiles indicate SR-Scores close to 1 (indicative of specific activation), and white tiles indicate unspecific activation. Gray boxes indicate no detectable effects at the highest tested concentration. SR_cytotoxicity_ was preferred over SR_baseline_. SR_baseline_ was used when no experimental IC_10_ values were available. Asterisks mark cases where SR or SR-Scores were calculated using IC_10, baseline_. (c) Two dimensions of toxicity of the BPA alternatives: Relative Effect Potency for cytotoxicity, REP_BPA, IC10_, median of all cell types versus the Cumulative SR-Score (Σ-SR-Score). BPA is highlighted in pink, BADGE in yellow, and the TMCDs in green. Data are provided in Table S6.

Our results align well with those reported in the literature (details in Text S5.4 and Tables S2 and S7).^16,25,26,57–60^ For instance, BPAF and BPZ are consistently reported as the most potent alternatives (REP_BPA,Erα_ = 3–5) and BP-MIBK as a highly potent impurity (REP_BPA,ERα_ = 8.5),^61^ while BPS is often mentioned as less estrogenic than BPA (REP_BPA, ERα_ = 0.02–1).

Very bulky substitutions at the para- and ortho-positions of the phenol rings, as seen in BPPH, TMBPF, TBBPA, BADGE D8, BPS-MAE, and BPS-MPE, were observed to suppress the ERα agonistic activity. Steric hindrance at the phenol ring or the inability to stabilize the active conformation of the receptor might explain this observation.^62,63^ TMBPF was also nonestrogenic in vivo and had therefore been proposed by Soto et al.^29^ as a safer BPA alternative for epoxy resins. The hydroxyl group positioning appears to be important for receptor activation, as seen in 2,4-BPS, which was 10 times less potent than BPS.^16,58^

In summary, structurally close BPA alternatives remained potent ERα agonists. Minor structural modifications, particularly at the methylene bridge, did not reduce estrogenic activity—some even resulted in the opposite. Those BPA alternatives with the closest structural resemblance to BPA also mimicked most closely its toxicological activity pattern/profile. We identified promising candidates that meet the criterion of eliminated ERα activation such as TMBPF, TMCD, BPS-MPE, Pergafast, and BTUM.

#### 3.5.2 Peroxisome Proliferator-Activated Receptor γ (PPARγ)

Many of the BPA alternatives with reduced ERα activation emerged as highly potent PPARγ agonists, while BPA and most structurally related BPA alternatives in Group III did not activate PPARγ at concentrations up to IC_10_ (Figure 4a). Among the active congeners, TBBPA was the strongest agonist in the PPARγ assay with an EC_10_ of 85 nM, which is only 340× higher than the reference compound rosiglitazone (0.03 nM). PPARγ agonism was observed for 11 alternatives, mostly in the nanomolar range or slightly higher.

Since BPA did not activate PPARγ, we could not derive any REP_BPA_ as a comparative measure for PPARγ activation. Effect concentrations for comparison are provided in Table 1, Table S6, and Figure S7. All PPARγ agonists activated the receptor with high specificity, with TBBPA and BTUM exhibiting the highest SRs of 148 and 121, respectively (Figure 4a). As observed for ERα, all active agonists induced the PPARγ only partially, with a maximum response of 52% for TBBPA, followed by a maximum response of 32% for tetrachlorobisphenol A (TCBPA) and 47% for BTUM (Table 1).

We observed shared structural characteristics among the PPARγ agonists in our test set, either the presence of an ortho-substituted bisphenol structure (TBBPA, TCBPA, Bisphenol G (BPG), BPPH, and TMBPF) or the presence of a charged sulfonyl functional group along with bulky para substituents (BPS-MPE, BPS-MAE, Pergafast, and BTUM).

The interaction of halogenated bisphenols with PPARγ was investigated thoroughly by Riu et al.^64,65^ They hypothesized that the large halogen atoms enhanced the receptor–ligand complex stability via additional van der Waals interactions. Other *ortho*-substituted bisphenols, such as BPG, BPPH, and TMBPF may exhibit their effects via a similar mechanism.^66^

Two studies tested various BPS derivatives, Pergafast, and BTUM for both PPARγ transactivation and the ability to induce adipogenesis in mouse 3T3-L1 cells.^67,68^ They reported significant PPARγ activation for BPS-MPE in the micromolar range, with weaker effects observed for D8, Pergafast, BPS-MAE, and BTUM. No agonism was detected for BPAF, BPA, 2,4-BPS, or BPB. Consistent with our findings, they observed a shift in receptor selectivity from ERα toward PPARγ, with the most adipogenic bisphenols, e.g., TMBPF, being inactive in estrogenicity assays using MCF-7 cells.^58,69^

Few environmental contaminants, such as phthalates (MHEP) and organotins, are known PPARγ activators, primarily at micromolar concentrations.^64^ In contrast, several bisphenol alternatives in this study (e.g., TBBPA, BTUM, and BPS-MPE) activated PPARγ at nanomolar concentrations. PPARγ agonists have been linked to obesogenic effects.^70^ Overall, these findings emphasize the need to test for more than just the absence of similar toxicological profiles and to anticipate newly emerging adverse effects.

#### 3.5.3 Mitochondrial Membrane Potential (MMP) Inhibition

Similar to PPARγ activation, mitochondrial membrane potential (MMP) inhibition does not appear to be a relevant toxicological end point for BPA itself, as it was a relatively weak MMP inhibitor (EC_10_ = 28 μM, Figure 3 and Table 1) with low specificity (SR = 3, Figure 4a). However, several BPA alternatives exhibited moderate MMP inhibition with high specificity (SR > 10) such as BPS and all its derivatives, BPP and BPT, as well as benzyl 4-hydroxybenzoate (BzPB). MMP inhibition was most pronounced for BPA alternatives with para-substituents and deprotonation under physiological conditions, e.g., D8 (SR = 41, 61% neutral, 39% anionic) and BPS-MPE (SR = 22, 68% neutral, 32% anionic) (Figure 1).

This is consistent with the mechanism of protonophoric uncoupling, which is pH-dependent^71^ and enhanced by the presence of electron-withdrawing groups, bulky hydrophobic moieties, and dissociable hydroxyl groups.^72^ The sulfonyl and ester groups as present in BPS derivatives and BzPB act as electron-withdrawing groups that decrease the p*K*_a_ and thus promote the uncoupling activity compared to BPA and structurally similar alternatives that are predominantly neutral under physiological conditions and do not shuttle protons.^73^

#### 3.5.4 Oxidative Stress Response (ARE)

Mitochondrial toxicity and oxidative stress responses are interrelated processes, as mitochondrial dysfunction can lead to increased reactive oxygen species production, which in turn exacerbates oxidative stress.^74,75^ Nevertheless, oxidative stress induction, as measured by ARE activation, does not appear to be a relevant primary toxicity end point for BPA and its alternatives (Table 1 and Figure 4a). Only D8 and BzPB exhibited a weak oxidative stress response exceeding an induction ratio of 1.5, but with low specificity (SR < 10). In agreement with previous findings,^76^ BPA did not induce ARE. BADGE lacked any oxidative stress response, despite containing reactive epoxide groups, likely due to hydrolysis to diols (BADGE-H_2_O) or reactions with proteins in the cell culture medium before cellular uptake.^51^

#### 3.5.5 Neurite Outgrowth Inhibition (NOI)

BPA alternatives also interfered with neurite outgrowth,^75^ a key process in neurodevelopment. BPA and 15 BPA alternatives inhibited neurite outgrowth with moderate specificity (SR < 10) in SH-SY5Y (Figure 4a and S7b). We could not confirm the finding of BPA or any of its proposed alternatives having a proliferative effect on SH-SY5Y at lower doses after 24-h exposure.^77^ Our results also contradict observed low-dose effects reported by Liang et al.,^78^ who found a significant reduction in average neurite length in neuronal stem cells for BPA, BPS, and BPF already at concentrations of 1 nM.

#### 3.5.6 Aryl Hydrocarbon Receptor (AhR)

AhR was activated by BPA and five alternatives but not beyond SR of 10 (Figure 4a), which is consistent with the literature, where insignificant AhR activation was reported for BPS^79^ and other bisphenols, with the exception of TGSA (bis­(3-allyl-4-hydroxyphenylsulfone).^16,80^

#### 3.5.7 Cumulative Specificity Ratio Score (Σ-SR-Score)

To account for activity at all end points, we defined the Cumulative SR-Score (eq 10). This novel metric measures a compound’s specific activity across the entire test battery, irrespective of the in vitro toxicological profile of the reference compound (Text S6, Table S6, and Figure 4b). This approach has several advantages compared to ToxPi,^81,82^ which is commonly used for integrating toxicological data: Σ-SR-Score ranks substances with broad toxicological profiles more highly, while ensuring that a single effect concentration or SR does not distort the overall score and ranking (Text S7, Table S8).

Most BPA alternatives, including BPA itself, exhibited specific activity in at least one assay (Figure 4). Both TMCDs and BADGE were not specifically active in all tested assays with cumulative SR-Scores <1, making them the only alternatives in this study without an identified specific mode of activation. BPA and several close structural analogues had a Cumulative SR-Score ≈1, indicating that they were specifically active for only one end point (mostly ERα) (Figure 4c).

Nine alternatives reached a Cumulative SR-Score close to 2, indicating that at least one effect is relevant. For group I, this was mainly due to the activation of PPARγ. We also observed compounds with activity in three or four assays, e.g., BzPB and BPS-MAE, which indicates higher promiscuity than BPA.

#### 3.5.8 Key Findings and Overall Interpretation of Bioassay Results

To summarize, EC_10_ and EC_IR1.5_ ranged over 6 orders of magnitude and the BPA alternatives activated at least one of the three nuclear receptors or affected the other end points at concentrations below IC_10_ (Table 1 and Figure S7a). BPA and its alternatives demonstrated the highest potency and specificity (SR_cytotoxicity_ > 10) for the PPARγ and ERα end points, while the other end points had an SR_cytotoxicity_ <10 or were not activated at all (Figure 4a). We also observed an inverse trend for BPA alternatives’ activity in ERα and PPARγ: those that lost their ERα activation due to structural modification often activated PPARγ, and the highly ERα-active BPA alternatives did not activate PPARγ.

For charged congeners, including BPS and its derivatives (Group IV), as well as the halogenated bisphenols, we also found specific mitochondrial membrane potential inhibition with SR_cytotoxicity_ > 10 coinciding with ERα activation. Neither oxidative stress, AhR activation, nor neurite outgrowth inhibition were identified as relevant end points for BPA and its alternatives. Racemic and trans-TMCD (Group V) were the least potent among the tested BPA alternatives and were not specifically active (Table S6).

An initial screening was also performed for the activation of p53 tumor suppressor regulation using the p53RE-*bla* HCT-116 cell line (Thermo Fisher) and the activation of the glucocorticoid receptor (GR) using the GR-UAS-*bla* HEK293T cell line (Thermo Fisher), but no activity above baseline cytotoxicity was observed for any compound. These assays were therefore not included in the final test battery.

Our findings demonstrate that many of the tested BPA alternatives are potent ERα agonists, with several even exceeding BPA’s activity. However, toxicity profiles of some candidates were markedly different for other end points, with some displaying greater promiscuity than BPA in the test battery. Remarkably, several alternatives emerged as highly potent PPARγ agonists, while some were moderate mitochondrial toxicants—end points irrelevant for BPA itself. These results highlight the need for a broader toxicological evaluation of BPA alternatives, particularly regarding metabolic and mitochondrial effects.

### 3.6 Abiotic CYP Assay Revealed BPA and Its Alternatives Are Oxidized, but No Highly Potent Metabolites Are Formed

To evaluate whether biomimetic catalyzed oxidation produces bioactive metabolites, we applied a novel high-throughput abiotic CYP (aCYP) assay.^34^ Two sets of experiments were run: First BPA and its alternatives were screened independently for reduction of parent concentration (Table S9) and changes in bioassay responses (Table S10) and in a second set of experiments, both measurements were conducted in parallel (Table S11).

Oxidation was highly structure-dependent, with substantial differences among the compounds (Table S9). *C*_parent_ of BPA was only reduced by 15% after oxidation, while the *C*_parent_ of BPT was reduced by more than 71% (Figure 5a). Bulky substituents at the methylene bridge or phenyl rings (group I) seem to impede the interactions with the catalytic center of TDCPP, resulting in low oxidation efficacy. However, there were also large variations of *C*_parent_-Ratio presumably due to the lower solubility of the very hydrophobic BPA alternatives, which might have led to some experimental artifacts due to precipitation. The sulfonyl amide structures (group II, BTUM 70%, Pergafast 44%) showed substantial loss by oxidation, while structures similar to BPA (group III) were either not oxidized or only moderately oxidized. Notable exceptions included the previously mentioned thioether BPT (71%) and BPF (48%), which both carry an unsubstituted bridge atom. Electron-withdrawing groups (−SO_2_) likely reduce susceptibility to oxidation, and accordingly, the entire group IV remained stable during aCYP oxidation. TMCD (group V) also remained unchanged, likely due to steric hindrance from its four methyl groups and the inherent stability of the substituted C_4_-ring.

**Figure 5:**
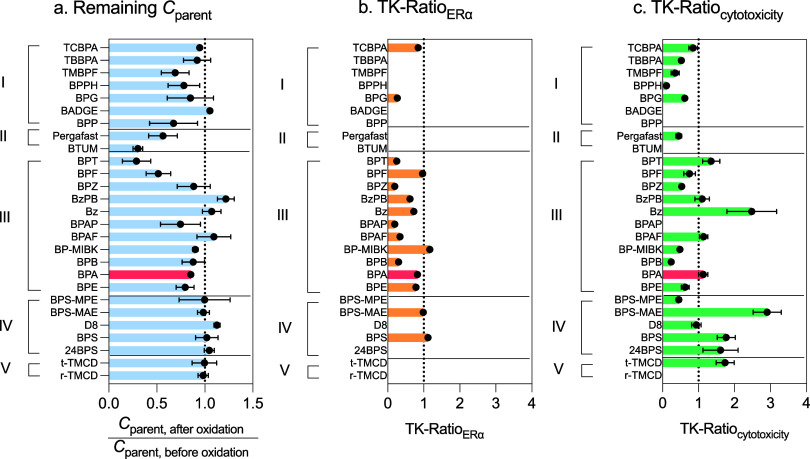
(a) Remaining parent compound (*C*_parent_-Ratio) after abiotic CYP oxidation, (b) TK-Ratio of ERα activity EC_10_, and (c) cytotoxicity (IC_10_). Results are grouped I–V by chemical similarity; error bars indicate error-propagated standard error for TK-Ratios (*n* = 2) and error-propagated standard deviation for *C*_parent_-Ratios (*n* = 2). BPA is highlighted in red. Data are provided in Tables S9 and S10.

For BPT we tentatively identified a degradation product (*m*/*z* = 235.0425; Text S8.1 and Figure S14), indicative of monohydroxylated BPT. Sulfoxidation of BPT to 4,4′-sulfinyldiphenol (SIDP) is a plausible metabolic pathway,^83^ though confirmation would require an analytical standard.

Estrogenic activity remained unchanged or decreased for BPA and all alternatives (Figure 5b and Table S10). Of the 26 BPA alternatives tested, 20, as well as BPA itself, showed similar or reduced cytotoxicity (TK_cytotoxicity_ ≤ 2) after abiotic CYP treatment (Figure 5c and Table S10). Bz (TK_cytotoxicity_ = 2.48 ± 0.69) and BPS-MAE (TK_cytotoxicity_ = 2.91 ± 0.39) exhibited a more than 2-fold increase in cytotoxicity (Figure 5c and Table S10), suggesting the formation of more cytotoxic metabolites. However, this increase in cytotoxicity was not accompanied by a reduction in the parent compound, which either hints at the formation of very toxic metabolites or suggests inaccuracies in the cytotoxicity IC_10_. More experiments would be required.

Due to their limited solubility, hydrophobic bisphenols are prone to solubilization artifacts, which may explain the observed variability in *C*_parent_-Ratios and underestimation of TK-Ratios. For instance, for BPAP 25% reduction of *C*_parent_ was accompanied by 80% reduction in estrogenicity (Figure 5). When the experiment was repeated with exposure and effect assessment in the same assay, *C*_parent_-Ratio was similar, and TK_ERα_-Ratio was only reduced by 56% (Text S8.2 and Table S11, Figures S15 and S16).

BTUM gave inconclusive results in both experimental setups (Tables S10 and S11). Given BTUM’s poor solubility in methanol, acetonitrile, PBS, and bioassay medium, we hypothesize that hydroxylated metabolites may exhibit increased solubility, contributing to their elevated toxicity compared to the parent compound itself.

The resistance of BPA and its alternatives to oxidative metabolism or the tendency for detoxification is consistent with reports in the literature. Kitamura et al.^24^ found that hydroxylated BPA metabolites exhibit significantly lower estrogenicity than BPA. This trend may extend to other alternatives, particularly those that are structurally similar to BPA (Group III). Indeed, several alternatives (e.g., BPB, BPAF, BPZ, BPS) were shown to form ortho-hydroxylated metabolites upon treatment with human and rat liver microsomes.^33,84^

Since aCYP exclusively simulates the oxidation step of phase I metabolism, future work should also aim to integrate models that account for phase II reactions to enable a more comprehensive assessment of the metabolic activation of BPA and its alternatives. However, phase II metabolites are always more hydrophilic conjugates that are typically much less potent and too hydrophilic and bulky to bind to any nuclear receptors. So far, we have not identified any potent metabolite formation.

### 3.7 BPA Alternatives Should Be Assessed with Similar Stringency as BPA

For a comprehensive comparative assessment, both the REP_BPA_ (Figure 3) and the Cumulative SR-Score of all BPA alternatives with BPA can be compared (Figure 4c). Those with equal or higher Cumulative SR-Score than BPA and REP_BPA,IC10_ >1 are clearly regrettable substitutions. There are few congeners with REP_BPA,IC10_ <1, but if that is accompanied by much higher SR-scores (BPS-MAE, BzPB, and BPT), the lower overall toxicity is overridden by more or additional specific effects.

In vitro effects of BPA have been consistently associated with adverse in vivo outcomes, such as increased uterine weight.^21,30,85^ Consequently, BPA alternatives that demonstrate comparable in vitro estrogenic activities, even with modest differences in potency, should be subjected to in vitro-based read-across or grouping, as they are likely to elicit the same in vivo effects as BPA. Therefore, in line with the precautionary principle, we propose that all BPA alternatives with comparable ERα activity profiles should be classified as endocrine disruptors and reproductive toxicants like BPA under CLP regulation for classification, labeling, and packaging. Such a step would automatically restrict these chemicals for use in food contact materials according to the new Regulation 2024/3190,^6^ unless explicitly authorized.

While most BPA alternatives’ specific activity and relative potency indicate potentially similar hazards, a few compounds show a distinctly favorable profile. Both TMCDs appear to be the only promising candidates to replace BPA as a monomer in plastics, given their low REP_BPA, IC10_ and Cumulative SR-Score of zero (Figure 4c). TMCD does not have the same physiochemical properties and similar structure as BPA and is therefore not a 1:1 replacement like BPS or BPF. From a hazard perspective, it should be a much safer chemical with much lower potency and without identified specific modes of action. As the test battery was derived, it could be too limited to identify reactive toxicity beyond the oxidative stress response, e.g., genotoxicity. As EFSA had previously categorized TMCD as nongenotoxic based on in vitro and in vivo data,^86^ it is unlikely that TMCDs exhibit reactive toxicity, and receptor-mediated toxicity is also unlikely due to its small size.

While TMCD itself might pose a low hazard potential, it is only one monomer of several in the commercialized plastics, which are very complex materials. The inactivity of a single monomer does not necessarily result in a safe plastic product. Oligomers might also still show harmful activity if leached out of the plastic.^87^ BADGE, a reactive prepolymer of BPA used for the coating of food containers, also fared quite well concerning the absence of specific effects and even low REP_BPA,IC10_ (Table S6). However, BADGE is unstable in aqueous solution, and therefore these results and conclusions should be treated with caution.

As TMCD has no dissociable protons, it cannot be applied to the second most important industrial application of BPA—as a color developer in thermal paper receipts (Figure 1b and Table S1). All alternatives that are suitable for this application exhibited specific activity for at least one of the tested end points (Cumulative SR-Score ≥ 1), mostly PPARγ, MMP, or ERα. This includes BPS-MPE, TMBPF, BPS-MAE, BzPB, BTUM, and Pergafast.

Many previous studies and reviews have focused on BPA and its alternatives. What has been lacking so far is a comprehensive data set and a comparative hazard assessment approach, so that potential BPA alternatives can be regulated as groups rather than individually. In the future, the Cumulative SR-Score in combination with the REP_BPA_ could serve as a practical screening tool for identifying suitable chemical alternatives based on specific bioactivity profiles. It could even be extended to additional end points or other metrics, such as TR and TK-Ratio or coupled persistency/biodegradation assays.

The TDI of BPA has just recently been substantially lowered to 0.2 ng/kg/day^88^ due to low-dose effects in vivo.^3^ We cannot find any reason why compounds with a similar toxicological profile, expressed as REP_BPA_ and Cumulative SR-Score ≥1, should be regulated differently merely because of missing in vivo data. In contrast, until proven otherwise, they should be treated as toxicological analogues and assessed as groups.

## Supplementary Material





## Data Availability

The concentration-response curves of all chemicals and bioassays are available at 10.5281/zenodo.16762455.

## Notes

Information on the experiments (acidity constant and octanol–water partition constant measurements, details of bioassay experiments and chemical analysis, detailed models. Details of similarity cluster analysis, additional evaluations (efficacy analysis, detection of metabolites), and figures, including heatmaps (PDF)
Detailed information on the chemicals, including physicochemical properties and predicted baseline toxicity, detailed toxicity results, SR, TR, TK-Ratios, SR-Scores, comparisons with literature (XLSX)
