# Supplementary material for: Beyond Estrogenicity: A Comparative Assessment of Bisphenol A and Its Alternatives in In Vitro Assays Questions Safety of Replacements
Source: Environ Sci Technol. 2025 Aug 16;59(33):17457–70. doi: 10.1021/acs.est.5c07018 (PMC12392461; doi:10.1021/acs.est.5c07018)
Supplement: Supplementary file 1 [file es5c07018_si_001.pdf]

Supporting Information

Beyond Estrogenicity: A Comparative Assessment of Bisphenol A and its Alternatives in In Vitro Assays Questions Safety of Replacements

Vanessa Srebny[†], Luise Henneberger[†], Maria König[†], Julia Huchthausen[†], Jenny Braasch[†] and Beate. I. Escher[†‡*]

[†]Department of Cell Toxicology, Helmholtz Centre for Environmental Research – UFZ, Permoser Str. 15, 04318 Leipzig, Germany

[‡]Environmental Toxicology, Department of Geosciences, Eberhard Karls University Tübingen, Schnarrenberger Str. 94-96, 72076, Tübingen, Germany‡

* Corresponding author email address beate.escher@ufz.de

Table of content

7 Additional texts (this file)
16 Figures (Figure S1 to S16–this file)
17 Tables (Table SA to SF–this file; Tables S1 to S11–XLSX file)

Tables in separate EXCEL

Table S1. BPA alternatives, their speciation at pH 7.4, liposome-water partition constant at pH 7.4 and predicted baseline cytotoxicity $IC_{10,baseline}$ for cell assays.

Table S2. Re-evaluated Tox21 Data (Adamovsky et al 2024), Relative Effect Potency (REP_{BPA}) and Specificity Ratio ($SR_{baseline}$).

Table S3. Bioassays including information on cell lines, reference compounds, cell culturing conditions.

Table S4. Assay medium composition and cell composition.

Table S5. All descriptors of the concentration-response curves.

Table S6. Cytotoxicity and effects of BPA alternatives and relative effect potency (REP) in comparison to BPA. Toxic ratios (TR) specificity ratios (SR) against experimental cytotoxicity and baseline cytotoxicity, Cumulative SR-Scores.

Table S7. Comparison of EC values, relative estrogenic potency (REP_{BPA}), and efficacy from various studies in literature.

Table S8: ToxPi scores according to Reif et al. and comparison of the resulting ranks with those obtained from the Cumulative SR-Score

Table S9. C_{Parent}-Ratio data of two replicate experiments and their mean.

Table S10. Inhibitory (IC_{10}) and effect concentrations (EC_{10}) before and after oxidation with the abiotic CYP catalyst. TK-Ratio for cytotoxicity ($TK_{cytotoxicity}$-Ratio) and effects (TK_{effect}-Ratio). Experiment without simultaneous measurement of c_{parent} concentrations.

Table S11. Inhibitory (IC_{10}) and effect concentrations (EC_{10}) before and after oxidation with the abiotic CYP catalyst. TK-Ratio for cytotoxicity ($TK_{cytotoxicity}$-Ratio) and effects (TK_{Effect}-Ratio). Simultaneously measured C_{parent} concentrations are reported as C_{parent} -Ratio.

S1: Test chemicals

S1.1: Chemical Structures of BPA and its alternatives

All BPA alternatives and BPA had been sourced from Chiron AS (Trondheim, Norway).

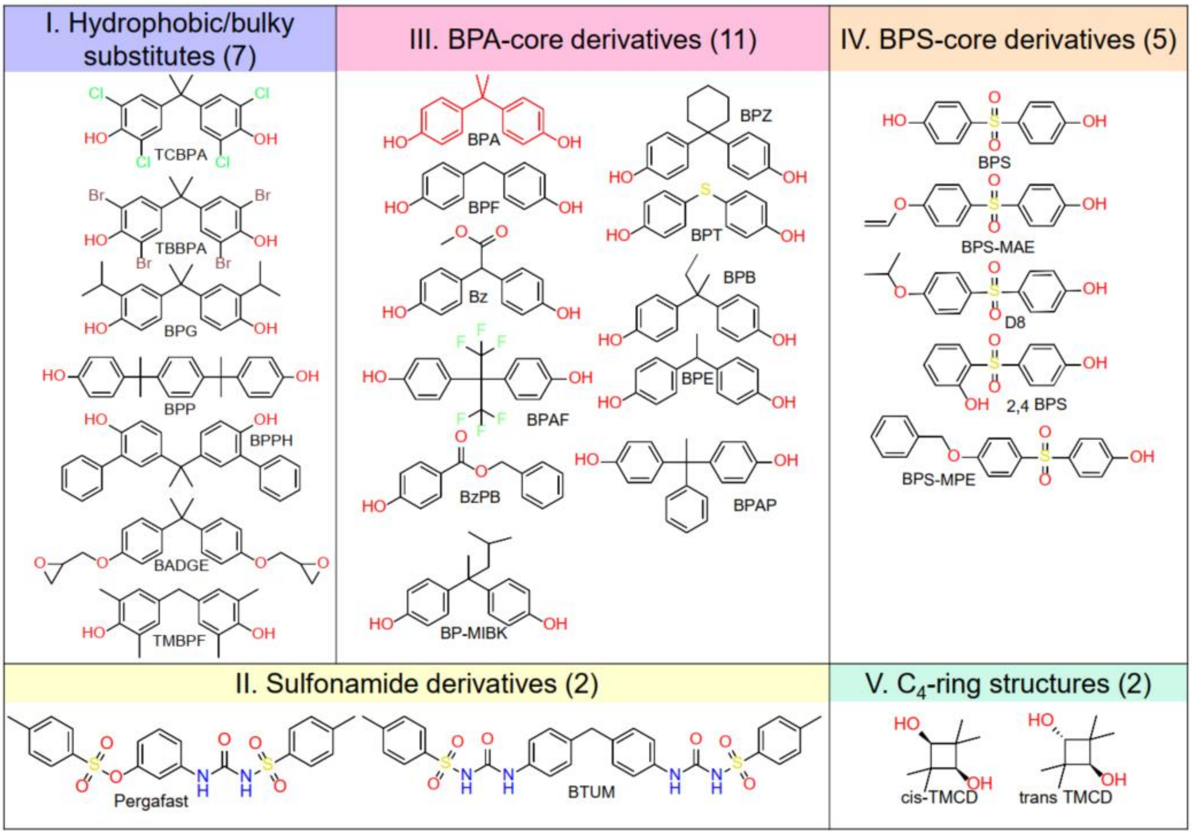

Figure S1: Chemical structures and hierarchical clustering of the molecular fingerprints of the 26 BPA alternatives. Group I: Hydrophobic/bulky substituents (blue), Group II: Sulfonamide derivatives (yellow), Group III: BPA-core derivatives (red), IV. BPS-core derivatives (orange), and V. C_4-ring structures (green). For readability, only abbreviations are shown in the figure. The full names are given in Table SA below and more details about the chemicals is in Table S1.

Table SA: Chemical names, abbreviations and CAS-Numbers of the tested chemicals.

Chemical name	Abbreviation	Short abbre-viation	CAS-Number
2,2',6,6'-Tetrachlorobisphenol A	Tetrachlorobisphenol A	TCBPA	79-95-8
3,3',5,5'-Tetrabromobisphenol A	Tetrabromobisphenol A	TBBPA	79-94-7
Bis(3,5-dimethyl-4-hydroxyphenyl)methane	Tetramethyl Bisphenol F	TMBPF	5384-21-4
2,2-Bis(2-hydroxy-5-biphenylyl)propane	Bipshenol PH	BPPH	24038-68-4
2,2-Bis(4-hydroxy-3-isopropylphenyl)propane	Bisphenol G	BPG	127-54-8
Bisphenol A diglycidyl ether	BADGE	BADGE	1675-54-3
4,4'-[1,4-Phenylenebis(1-methylethylidene)] bis-phenol	Bisphenol P	BPP	2167-51-3
N-(p-toluenesulfonyl)-N'-(3-(p-toluenesulfonyloxy)phenyl)urea	Pergafast 201	Pergafast	232938-43-1
4,4'-Bis(p-tolylsulfonylureido)diphenylmethane	BTUM	BTUM	151882-81-4
4,4'-Thiodiphenol	BPT	BPT	2664-63-3

Chemical name	Abbreviation	Short abbre-viation	CAS-Number
Bis(4-hydroxyphenyl)methane	Bisphenol F	BPF	620-92-8
1,1'-Bis(4-hydroxyphenyl)cyclohexane	Bisphenol Z	BPZ	843-55-0
Benzyl 4-hydroxybenzoate	Benzylparaben	BzPB	94-18-8
4-hydroxy-alpha-(4-hydroxyphenyl)-, methyl ester	Benzeneacetic acid	Bz	5129-00-0
4,4'-(1-Phenylethylidene)bisphenol	Bisphenol AP	BPAP	1571-75-1
4,4'-(1,1,1,3,3,3-Hexafluoropropane-2,2-diyl)diphenol	Bisphenol AF	BPAF	1478-61-1
4,4'-(1,3-Dimethylbutylidene)diphenol	Bisphenol-MIKB	BP-MIBK	6807-17-6
4,4'-(Butane-2,2-diyl)diphenol	Bisphenol B	BPB	77-40-7
4,4'-(Propane-2,2-diyl)diphenol	Bisphenol A	BPA	80-05-7
4,4′-Ethylidenbisphenol	Bisphenol E	BPE	2081-08-5
4-[4-(benzyloxy)benzenesulfonyl]phenol	Bisphenol S-MPE	BPS-MPE	63134-33-8
4-[[4-(Allyloxy)phenyl]sulfonyl]phenol	Bisphenol S-MAE	BPS-MAE	97042-18-7
4-((4-Isopropoxyphenyl)sulfonyl)phenol	D-8	D8	95235-30-6
4,4'-Sulfonyldiphenol	Bisphenol S	BPS	80-09-1
2,4'-Dihydroxydiphenyl sulfone	2,4-Bisphenol S	2,4-BPS	5397-34-2
trans-2,2,4,4-Tetramethyl-1,3-cyclobutanediol	trans -TMCD	t-TMCD	
2,2,4,4-Tetramethyl-1,3-cyclobutanediol (racemate)	racemic TMCD	r-TMCD	3010-96-6

S1.2: pK_a and log K_{ow} measurements

The acidity constants (pK_a) of 24 BPA alternatives and BPA were assessed using a Sirius T3 titrator (Pion Inc., Sussex, U.K.). A detailed description of the experimental procedure is available in the literature.[1] In brief, each sample was prepared by mixing 5 µL of a 10 mM dimethyl sulfoxide (DMSO) stock solution with 25 µL of a phosphate buffer solution (14.4 mM K_2HPO_4 and 0.15 M KCl) in a Sirius T3 test vial. A reference vial containing 5 µL of DMSO (Roth, A994-100 ML) and 25 µL of phosphate buffer was analyzed alongside each sample. The pK_a values were determined using the automated UV-metric pKa protocol within the Sirius T3 Control software (version 2.0.0.0.). Each sample was subjected to three consecutive titrations under a constant ionic strength of 0.15 M potassium chloride, covering a pH range from 2 to 12 by stepwise addition of 0.5 M HCl or 0.5 M potassium hydroxide while recording UV absorbance. For log K_{ow} measurement, approximately 1 mg of test chemical was added to a sample vial and the measurement was done automatically via the potentiometric log P protocol. Briefly, each compound is titrated thrice in an immiscible biphasic system of octanol and phosphate buffer solution and pH is continuously recorded. The shift in pK_a -values was used to estimate the log K_{ow} values.[2, 3] Data processing was performed with Sirius T3 Refine software (version 2.0.0.0.), and the final pK_a and log K_{ow} values were calculated as the average of three titrations. To ensure reproducibility, measurements were conducted on at least two separate days.

S1.3: Estimation of distribution ratios between biomaterials and water and baseline toxicity model

The ionization-corrected distribution ratios between biomembranes (liposomes) and water D_{lipw} can be estimated using the fraction of neutral species ($\alpha_{neutral}$) and the liposome-water partitioning constant (K_{lipw}) for neutral chemicals (eq. S1). This assumes that charged chemicals have approximately 10 times lower affinity to biological membranes.[4] K_{lipw} was derived from the octanol-water partitioning constant log K_{ow} (eq. S2).[5] This equation is only valid for log K_{ow} values between 1 and 8. D_{BSAw} for the bisphenols can be predicted with eq. S3 based on fraction α and log K_{ow} which is derived from combining eq. S4 and S5 which were developed for neutral compounds and anionic per- and polyfluoroalkyl substances (PFAS) respectively.[5-7] Analogous models are used for structural proteins (eq. S6 to S8).[6, 8]

$$D_{lip/w}\ (\text{pH } 7.4) = K_{lip/w,neutral} \times [\alpha_{neutral} + 0.1 \times (1-\alpha_{neutral})] \quad \text{(S1)}$$

$$\log K_{lip/w} = 1.01 \times \log K_{ow} + 0.12 \quad \text{(S2)}$$

$$\log D_{BSA/w}\ (\text{pH } 7.4) = \log (\alpha_{neutral} \times 10^{0.71\times \log K_{ow} + 0.42} + (1-\alpha_{neutral}) \times 10^{0.75 \times \log K_{ow} + 1.01} \quad \text{(S3)}$$

$$\log K_{BSA/w,neutral} = 0.71 \times \log K_{ow} + 0.42 \quad \text{(S4)}$$

$$\log D_{BSA/w,anion} = 0.75 \times \log K_{ow} + 1.01 \quad \text{(S5)}$$

$$\log D_{SP/w}(\text{pH } 7.4) = \log (\alpha_{neutral} \times 10^{0.72 \times \log K_{ow} - 0.47} + (1-\alpha_{neutral}) \times 10^{0.0.46 \times \log K_{ow} + 1.51} \quad \text{(S6)}$$

$$\log K_{SP/w,neutral} = 0.72 \times \log K_{ow} - 0.47 \quad \text{(S7)}$$

$$\log D_{SP/w,anion} = 0.46 \times \log K_{ow} + 1.51 \quad \text{(S8)}$$

A mass balance model can be set up to describe the binding to all compartments and to relate the critical membrane concentration for cytotoxicity $IC_{10,membrane}$ to the required nominal concentration in the medium ($IC_{10,\ nom,\ baseline}$). As the baseline toxicity model is based on distribution ratios in volume units, we converted literature data into mass units with a density of lipids of 1 kg_{lipid}/L_{lipid} and a density of proteins of 1.36 $kg_{protein}/L_{protein}$. For improved readability we have shortened $IC_{10,\ nom,\ baseline}$ to $IC_{10,\ baseline}$ in the remaining text.

$$IC_{10,nom,baseline}\ (M) = \frac{IC_{10,membrane}}{D_{lip/w}} \times \Big(1 + D_{BSA/w} \times VF_{protein,medium} + D_{lip/w} \times VF_{lipid,medium} + D_{SPw} \times VF_{protein,cell} + D_{lipw} \times VF_{lipid,cell}\Big) \quad \text{(S9)}$$

As $V_w \approx V_{tot}$, $VF_{protein,lipid} >> VF_{cell,lipid}$ and $VF_{protein,medium} >> VF_{cell,medium}$, eq. S9 can be simplified to (eq. S10), which is eq. 1 in the main manuscript with an $IC_{10,membrane}$ of 0.026 M.[9]

$$IC_{10,nom,baseline}(M) = \frac{IC_{10,membrane}}{D_{lip/w}} \times \Big(1 + D_{BSA/w} \times VF_{protein,medium} + D_{lip/w} \times VF_{lipid,medium}\Big) \quad \text{(S10)}$$

S1.4: Stability of BPA and its alternatives in PBS.

We tested the stability of all BPA alternatives and BPA in 0.1mM PBS (pH 7.4) for 48h at 37° C except for TMCD prior to the bioassay experiments. The experiment was performed as follows: 500 µL of a 2 mg/L solution of the chemical in PBS was aliquoted into 3 HPLC vials (n=3) and incubated for 48 h at 37 °C. The 2 mg/L solution was prepared by diluting the respective 10 g/L stock solution in a 10 mL glass crimp vial with an aluminum cap. For HPLC analysis, 500 µL acetonitrile was added prior to injection.

Most bisphenols were stable, and the concentrations only changed negligibly. BADGE undergoes rapid hydrolysis due to its epoxy groups,[10] while N-(p-toluenesulfonyl)-N'-(3-(p-toluene sulfonyloxy)phenyl)urea (Pergafast) was described to hydrolyze to methyl N-(p-tosyl) carbamate (MTC) and m-aminophenyl tosylate (AMT) under mildly acidic conditions (pH<7).[11]

We have not tested stability of TMCD in aqueous media because we lacked a suitable analytical procedure. However, TMCD has been reported to remain stable for 22 days and has shown no degradation in OECD guideline test 301B which is done in aqueous solution.[12]

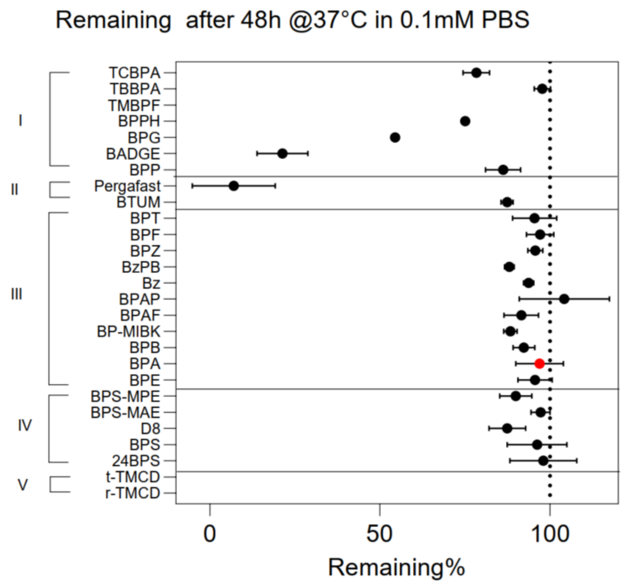

Figure S2: Detection of Bisphenol alternatives after 48 h incubation in 0.1mM PBS pH 7.4**.** Each data point represents the mean percentage with error bars indicating standard error of mean (SEM) of n=3 replicates.

S2: Cell culture and bioassays

S2.1: Cell Culturing Conditions

All suppliers and sources are given in detail in SI-Excel S3. The bioassay media for the different assays were prepared as follows: Assay medium for ER alpha-UAS-bla GripTite (ERα) and PPAR gamma-UAS-bla 293H (PPARγ) was prepared with 98% Opti-MEM, supplemented with 2% charcoal-stripped FBS, and 100 U/mL penicillin-streptomycin. MitoOxTox and AhR assay medium was comprised of 90% DMEM with GlutaMAX, supplemented with 10% FBS, and 100 U/mL penicillin-streptomycin. Neurotoxicity assay medium consisted of the neurobasal

medium, supplemented with 2% B-27 Supplement and 2 mM GlutaMAX Supplement. SH-SY5Y cells were differentiated with 10 µM all-*trans* retinoic acid prior to the assay.

S2.2: Cell Seeding and Incubation. For all bioassays, cell suspensions in the respective assay medium were dispensed into each well of 384-well plates using a MultiFlo Dispenser (Biotek, (now Agilent), Winooski, VT, U.S.). After seeding, the plates were incubated at 37° C and 5% CO_2 for 24° h. The final cell densities and plate types were as follows: Black poly-D-lysine coated 384-well plates (Product No. 354663, Corning Inc., NYC, U.S.) were used for PPARγ: 6500 cells/well, ERα: 5000 cells/well. White 384-well plates with a clear bottom (Product No. 781098, Greiner, Kremsmünster, Austria) were utilized for the MitoOxTox assay: 2650 cells/well and white poly-D-lysine coated 384-well plates (Product No. 354660, Corning Inc., NYC, U.S.) for AhR: 3250 cells/well. Black Collagen I coated plates were used (Product No. 356667, Corning Inc., NYC, U.S.) for SH-SY5Y cells: 3100 cells/well.

S2.3: Chemical Dosing and Assay Analysis Procedures. The dosing procedure and high-throughput assay workflow have been described in detail before.[9] The dosing concentrations for each chemical were estimated as 3× $IC_{10,baseline}$ (Table S1). Stock solutions were then either added directly to the respective cell culture medium or evaporated under nitrogen, followed by replenishment with fresh medium. The final solvent content in each well was kept below 1% for methanol, 0% for acetonitrile.

Dosing plates were prepared as 11-step serial dilutions using a Hamilton Microlab Star robotic system (Hamilton, Bonaduz, Switzerland), and chemicals were dosed in duplicates by transferring 10 µL twice from the dosing plates to the cell plates. After quantifying cell confluency as described in S2.4., the exposed cells were incubated at 37° C and 5% CO_2 for an additional 24 h. All chemicals were tested in all assays with at least three independent replicates.

S2.4: Cell Viability Measurements and bioanalytical protocols. The confluency as a measure of cell viability was determined with an IncuCyte S3 Live-Cell Analysis System (Sartorius AG, Essen, Germany). With this method the confluency of cells in a microscopy image are normalized to control wells without cells and compared before and after 24 hours of exposure. This method has been described previously[13]. Cell viability of SH-SY5Y cells was measured via live-dead staining with Nuclear Green LcS1 (138904, Abcam, Cambridge, U.K.) and propidium iodide (PI, Abcam, Cambridge, U.K.).[14] Nuclear Green LCS1 is a membrane-permeable dye that stains the nuclei of all cells, whereas PI only enters cells with compromised membrane integrity (non-viable cells). After staining, live cells show green nuclei without red fluorescence, while dead cells display both green and red nuclear fluorescence due to PI

intercalation. Cell viability was calculated as the proportion of green-only nuclei relative to the total number of nuclei.

For experiments with the preceding aCYP activation, ERα cell viability was additionally calculated from the readout of the ToxBLAzer reagent. The ToxBLAzer assay was conducted following the manufacturer's instructions (Thermo Fisher, Waltham, MA, U.S.).[13] Fluorescence intensity was recorded at the start of the assay ($F_{wavelength}(0h)$) and after a 2-hour incubation at room temperature ($F_{wavelength}(2h)$) using three excitation/emission wavelength pairs: 409/460 nm (blue), 409/530 nm (green), and 590/665 nm (red).

Cell viability was assessed based on the red fluorescence signal at 665 nm, applying eq. S11 for normalization.

$$\text{Cell viability} = \frac{F_{665nm}(\text{2h, sample}) - F_{665nm}(\text{0h, average cell-free})}{F_{665nm}(\text{2h, average unexposed}) - F_{665nm}(\text{0h, average cell-free})} \quad (S11)$$

Only data with cell viability >90% (i.e., concentrations <IC_{10}) were included for evaluation of the specific endpoint to avoid artifacts from cytotoxicity burst.[15]

S2.5: Concentration-response curves (CRCs)

To avoid bias from cytotoxicity burst at higher concentrations, all concentrations exceeding the IC_{10} for cytotoxicity were excluded in the CRC modeling of effects but otherwise cytotoxicity and effects were fitted with the same model equations (response stands for cytotoxicity or effect). For ERα, AhR, ARE and PPARγ preferably a linear model was fitted to the linear portion of CRC up to maximum 30% effect (eq. S12),[16] but in some cases it was necessary to choose a sigmoidal log-concentration-response model, specifically a log-logistic model (eq. S13), to derive effect concentrations and cytotoxicity. For mitochondrial toxicity and neurotoxicity, the log-logistic model was always applied and no cytotoxicity IC_{10} cutoff was applied because these are not reporter gene assays. Common practice when using log-logistic models, e.g., the model implemented in tcpl package in R,[17, 18] is that the minima and maxima are also fitted and only median effect concentrations AC50 are derived.

In the present study, we had to calculate absolute 10% inhibitory (IC_{10}) or effect concentrations (EC_{10}) for both linear and log-sigmoidal CRCs to allow absolute comparisons at the same effect level and for TR and SR-analysis. This was done using the AutomatedBioassayScreening[19] published by Braun et al.,[20] which also included an algorithm to choose the best model between eq. S12 and eq. S13.

$$\text{Response (\%)} = \text{slope} \times C \quad (S12)$$

$$\text{Response (\%)} = \frac{100\%}{1+10^{\text{slope}(\log EC_{50} - \log C)}} \quad (S13)$$

$$\log EC_{10} = \log EC_{50} - \frac{1}{\text{slope}} \cdot \log\left(\frac{\max - \min}{y - \min} - 100\%\right) \quad (S14)$$

Detailed information on curve-fitting parameters of the CRCs is given in Table S5.

The inhibitory concentration causing 10% cytotoxicity (IC_{10}) and the effective concentration resulting in 10% of the maximum effect (EC_{10}) were calculated by dividing 10% of the response signal by the slope of the linear concentration-response curve (eq. S15 and S16).

For ARE the concentration required for an induction ratio of 1.5 ($EC_{IR1.5}$) was obtained from a linear CRC up to IR of 4 through the intercept at IR 1 (eq. S17).

$$IC_{10} = \frac{0.1}{slope} \quad (S15)$$

$$EC_{10} = \frac{0.1}{slope} \quad (S16)$$

$$EC_{IR1.5} = \frac{0.5}{slope} \quad (S17)$$

Efficacy, expressed as Emax%, represents the ability of a compound to stabilize the active conformation of the investigated receptor in reporter gene assays for nuclear receptors. The maximum efficacy was determined by independently fitting the monotonic upwards part of the CRC and extracting the maximum of the log-logistic concentration-response curve Emax% (eq. S18).

$$\text{Response (\%)} = \frac{Emax\%}{1+10^{slope(logAC50-logC)}} \quad (S18)$$

S3: Instrumental Analysis

S3.1 Liquid Chromatography Triple Quadrupole Mass Spectrometry (LC-MS/MS)

A high-performance liquid chromatography system (HPLC 1260 Infinity II from Agilent, Santa Clara, CA, U.S.) was used for the separation and quantification of reaction components. The system was equipped with a flexible pump, a multisampler, a column thermostat, a diode array detector (DAD) and an external valve drive to switch detection between a triple quadrupole mass spectrometer with ESI source and a fluorescence detector (FLD). Various detection methods were applied depending on the analyte properties. Preferably MRM methods were used (Table SA-B). 3,3',5,5'-Tetrabromobisphenol A (TBBPA) and Pergafast could only be detected with a DAD. 2,2-Bis(4-hydroxy-3-isopropylphenyl)propane (BPG) was detected via an FLD detector (Table SC). The chromatographic separation was performed using a Kinetex C18 LC column (particle size: 1.7 µm, column dimension: 50 × 2.1 mm) or a Luna Omega polar C18 LC column (particle size: 1.7 µm, column dimension: 50 × 2.1 mm). The mobile phase composition and gradient are described in Table SB-D.

The chromatographic separation of Pergafast and TBBPA was carried out using a gradient elution. The mobile phase consisted of Water (100%) as Solvent A and acetonitrile:water (95:5%) as Solvent B, with a constant flow rate of 0.5 mL/min. The gradient program began with 75% A and 25% B, which was maintained for 1 minute. From 1.00 to 5.00 minutes, the proportion of B increased linearly to 100%, where it was held for 0.5 minutes,

followed by a re-equilibration to initial conditions from 5.50 to 8.00 minutes. Detection was performed using a diode array detector (DAD), with signals acquired at 220 nm for Pergafast and 205 nm for TBBPA. Additionally, the column temperature was set to 40° C for Pergafast to optimize separation conditions.

S3.2 Gas chromatography Triple Quadrupole Mass Spectrometry (GC-MS/MS)

A gas chromatography system (GC 8890 from Agilent) coupled with a triple quadrupole mass spectrometer (7010B GC/TQ) was used for the analysis of both isomers of trans-2,2,4,4-Tetramethyl-1,3-cyclobutanediol (TMCD). The system was equipped with an autosampler from Gerstel (Mülheim an der Ruhr, Germany), a splitless inlet, and a helium carrier gas supply. Sample injection was performed with a volume of 1 µL at an inlet temperature of 260 C. The inlet flow was set to 54 mL/min, and an Agilent 5190-3162 liner (900 µL) was used. The chromatographic separation was achieved on an Agilent HP-5MS column (60 m × 0.25 mm × 0.25 µm).

The oven temperature program started at 100° C with a holding time of 5 min, followed by a ramp of 20 C/min to 320 C, which was held for 2 min. The total runtime was 18 min. Detection was performed in selected ion monitoring (SIM) mode with positive ionization. Target ions were m/z 72 and m/z 57, each monitored with a dwell time of 250 ms. The resolution was set to "wide."

S3.3 High-resolution Orbitrap liquid chromatography (LC-HRMS) for untargeted screening and structural elucidation of unknown reaction products

The eluents and gradient conditions were the same as in Braun et al.[21] Liquid chromatography-high resolution mass spectrometry (LC-HRMS) analysis was carried out using a Thermo Ultimate 3000 LC system, equipped with an electrospray ionization (ESI) source and an Orbitrap Exploris 480 mass spectrometer (Thermo Fisher Scientific, Waltham, MA, U.S.). The chromatographic separation was performed on an ACQUITY UPLC® BEH C18 column (100 x 2 mm, 1.7 µm particle size, Waters, Milford, MA, U.S.).

For positive ion mode, the mobile phase consisted of 1 mM ammonium formate (70221, Sigma Aldrich) with 0.1% formic acid (56302, Honeywell) in water (10728098, Thermo Fisher Scientific) and methanol (MeOH). In negative ion mode, 2 mM ammonium bicarbonate (A6141, Sigma) in water and a 95:5 MeOH:water mixture were used. The gradient program is detailed in Braun et al. 2024.[20] Solvent blanks contained a 10:90 water:MeOH ratio.

Sample preparation involved diluting the abiotic CYP mix to a final concentration of 1mg/L in water:MeOH 10:90 and adding 10 µL of an internal standard mixture (100 ng/mL). A 25 µL injection volume was used. The ion transfer capillary was heated to 325° C, while the vaporizer temperature was set to 350° C. The spray voltage was adjusted to 2.5 kV for positive

mode and -2.0 kV for negative mode. The sheath gas flow rate was maintained at 45 arbitrary units (a.u.), with an auxiliary gas flow rate of 7 a.u. in static mode. Internal mass calibration was performed using the EASY-ICTM system (Thermo Fisher Scientific). The eluents and gradient conditions were the same as in Braun et al. 2024.[20]

Separate analyses were conducted for positive and negative ionization modes. Each run included a full scan (m/z 80-1200) with a nominal resolving power of 120,000 at m/z 200, recorded in profile mode. Additionally, data-independent MS/MS experiments were performed with a resolving power of 45 000 in centroid mode. For fragmentation, broad isolation windows were applied, covering m/z 80-182, 180-282, 280-382, and 380-482 (100 mu), m/z 480-682 (280 mu), and m/z 680-1200 (500 mu).

The column oven temperature was maintained at 50° C, and the flow rate was set to 0.3 mL/min. An equilibration time of 8 minutes was applied before analysis.

Table SB: Liquid Chromatography Instrumental Settings with Formic Acid (FA) eluent method.

Compound	CAS	Flow (ml/min)	Solvents	Elution (A:B)	Retention time (min)	Column	MRM transitions (parent)	ESI +/-	MRM transitions (fragments)	Fragmen-tor	Collision Energy	Cell accele-rator voltage
TCBPA	79-95-8			40:60	1.1		362.9	negative	248,<br>210.1	200	40<br>20	4
D8	95235-30-6			55:45	0.9		291.1	negative	183.9<br>159.4	150	30<br>22	4
BzPB	94-18-8			65:35	2.1		227.1	negative	136.2<br>92.1	100	10<br>22	4
Bz	5129-00-0		0.5	75:25	1.1	Kinetex 1.7 µm, C18, 100 Å, LC column (50 × 2.1 mm)	257.1	negative	197	150	18	4
BPS-MAE	97042-18-7	0.5	(A) 5% ACN in water +0.1% FA; (B) 95% ACN in water + 0.1% FA	60:40	1.1		289	negative	248<br>184<br>108	100	14<br>30<br>40	4
BPS-MPE	63134-33-8			55:45	1.4		339.1	negative	248<br>184	150	18<br>34	4
BTUM	151882-81-4			50:50	1.5		591.1	negative	491.6<br>170	200	30<br>34	4
BPS	80-09-1			70:30	0.65<br>0.93	Luna Omega 1.7 µm, polar C18, LC column (50 × 2.1 mm)	249	negative	107.9<br>92	150	30<br>40	4
2,4- BPS	5397-34-2			70:30	0.86<br>1.1		249	negative	155.9<br>108<br>92	100	10<br>22<br>26	4

Table SC: Liquid Chromatography Instrumental Settings with Ammonium fluoride (AmF) eluent method.

Abbreviation	CAS	Flow (ml/min)	Solvents	Elution (A:B:C)	Retention time (min)	Column	MRM transitions (parent)	ESI +/-	MRM transitions (fragments)	Fragmentor	Collision Energy	Cell Accelerator Voltage
BPA	80-05-7	0.5	(A) 100% Water +1mM AmF; (B) 100% ACN (C) 100% MeOH + 1mM AmF	65:35:0	2	Kinetex 1.7 µm, C18, 100 Å, LC column (50 × 2.1 mm)	227.1	negative	212.4	150	14	4
BPAP	1571-75-1			55:45:0	2		289.1	negative	274.4	150	18	4
BPE	5081-08-5			65:35:0	2		213.1	negative	198.3<br>197.3	150	14<br>34	4
BPP	2167-51-3			47:53:0	2.5		364.8	positive	364.8	100	2	4
BPPH	24038-68-4			40:60:0	2.5		398.2	positive	398.9<br>211.4	100	2<br>6	4
BPZ	843-55-0			55:45:0	2.2		286.2	positive	175.3<br>175.3	100	6<br>38	4
BPB	77-407			55:45:0	2		241.1	negative	211.3	150	26	4
BP-MIBK	6807-17-6			50:50:0	2.5		269.2	negative	212.3	150	18	4
BPAF	1478-61-1			55:45:0	2		335	negative	265.4	150	22	4
BADGE	1675-54-3			45:55:0	2		358.2	positive	191.3<br>161.2<br>135.1<br>107.1	100	10<br>18<br>34<br>40	4
BPT	2664-63-3			55:45:	2.1		217	negative	124.1	100	18	4
TMBPF	5384-21-4			40:0:60:	2		274.2	Positive	135.1<br>91.2	100	40	4

Table SD: Liquid Chromatography Instrumental Settings with BPs only detected with DAD and FLD.

Abbreviation	CAS	Flow (ml/min)	Solvents	Elution (A:B)	Retention time (min)	Column	Detector	Excitation (nm)	Absorption (nm)
BPG	127-54-8	0.5	(A) 5% ACN in water +0.1% FA;<br>(B) 95% ACN in water + 0.1% FA	35:65	2	A Kinetex 1.7 µm,<br>C18, 100 Å,	FLD	223	320
TBBPA	79-94-7	0.5		Gradient	2	LC column (50 × 2.1 mm)	DAD	-	209
Pergafast	232938-43-1	0.5	(A) 5% ACN in water;<br>(B) 95% ACN in water	Gradient	5		DAD	-	220

Table SE: Internal Standard Mix for LC-HRMS

Internal Standard 1	Internal Standard 2
Mono-isobutylphthalate-D4	Decyltrimethylammonium-D30
Creatinine-D3	Atenolol-D7
Diazinon-D10	Progesterone-D9
Benzophenone-3-D5	Verapamil-D6
p-Toluene-sulfonamide-D4	Bezafibrate-D4
Cotinine-D3	Sulfamethoxazole-D4
Diglyme-D6	Tebuconazole-D9
Chlormequat-D9	Imidacloprid-D4
Carbamazepine-D10	4-Nitrophenol-D4
Atrazine-13C3	Triclosan-D3
Benzotriazole-D4	Mecoprop-D3
Carbendazim-D4	Acesulfame-D4
Tri-n-butylphosphate-D27	Hydrochlorothiazide-13C6
DEET-D7	Bentazone-D6
Metolachlor-D6	Cyclamate-D11
Isoproturon-D3	Clarithromycin-D3
Diclofenac-D4	Desisopropylatrazine-D5
Caffeine-D3	

S4: Chemical Fingerprint Similarity Clustering

Four different hierarchical clustering methods—single, complete, average, and Ward linkage—were explored to identify the optimal approach for grouping the molecules. The clustering results were visualized as dendrograms (Figure S3) using the stats and ggdendro packages. The cut-off for the grouping was chosen as 1.25. Principal Component Analysis (PCA) (Figure S4) and Silhouette scores (Figure S5) provided a measure of cluster quality. 4,4'-[1,4-Phenylenebis(1-methylethylidene)] bis-phenol (BPP) was not successfully grouped by the clustering algorithm, because its fingerprint contains BPA twice as a substructure. Due to its high molecular weight, hydrophobicity, and bulkiness we manually assigned it to Group I.

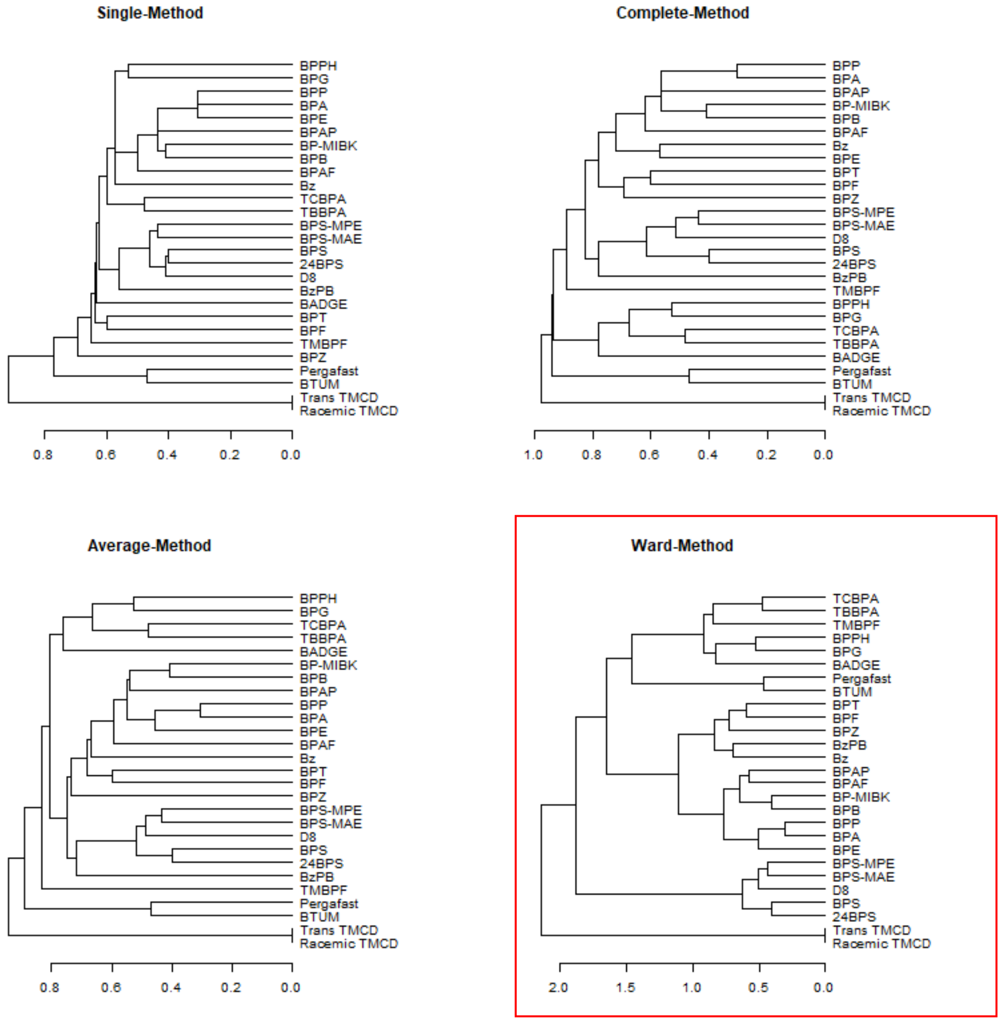

Figure S3: Cluster Quality Metrics for Fingerprint and Linkage Combinations (Dendrograms). (a) Single Method, (b) Complete -Method, (c) Average- Method and (d) Ward-Method.

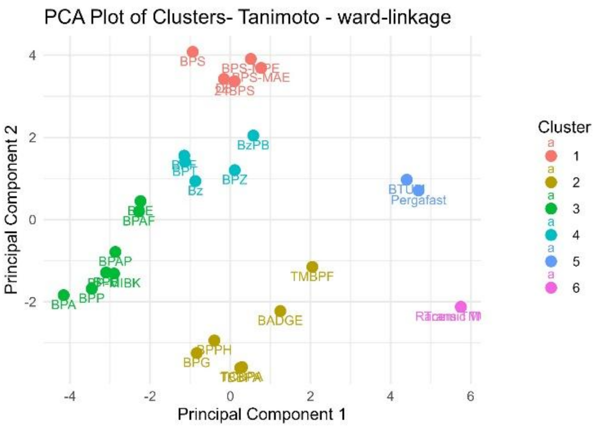

Figure S4: Chemical fingerprints analyzed with PCA, colored with clusters obtained with Ward-linkage.

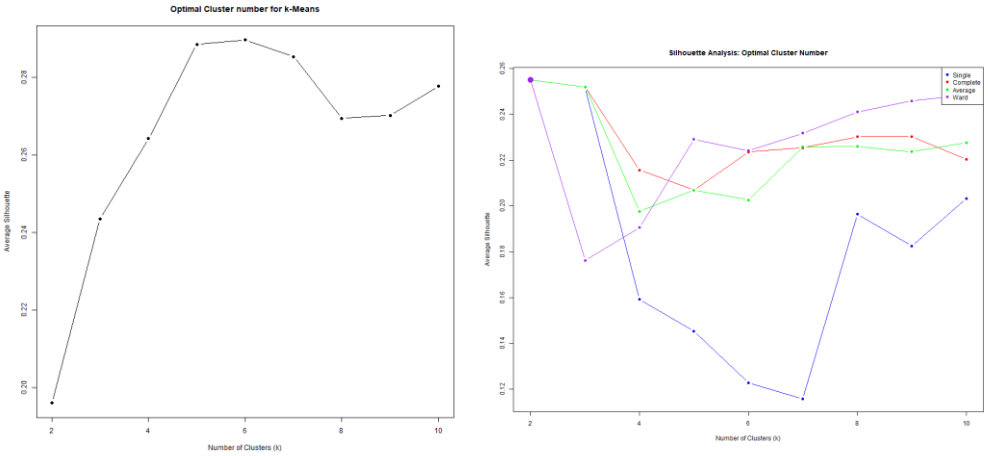

Figure S5: (a) Silhouette -Scoring for k-means with different number of cluster numbers (b) Silhouette -Scoring for different Clustering Methods (Single-, Complete-, Average-, Ward-Linkage).

Table SF: Silhouette -Scoring of Clustering Methods

Linkage Method	Single	Complete	Average	Ward	k-Means
Silhouette - Score	0.19	0.27	0.27	0.29	0.29

S5: Bioassay Results

S5.1 Median Cytotoxicity plots

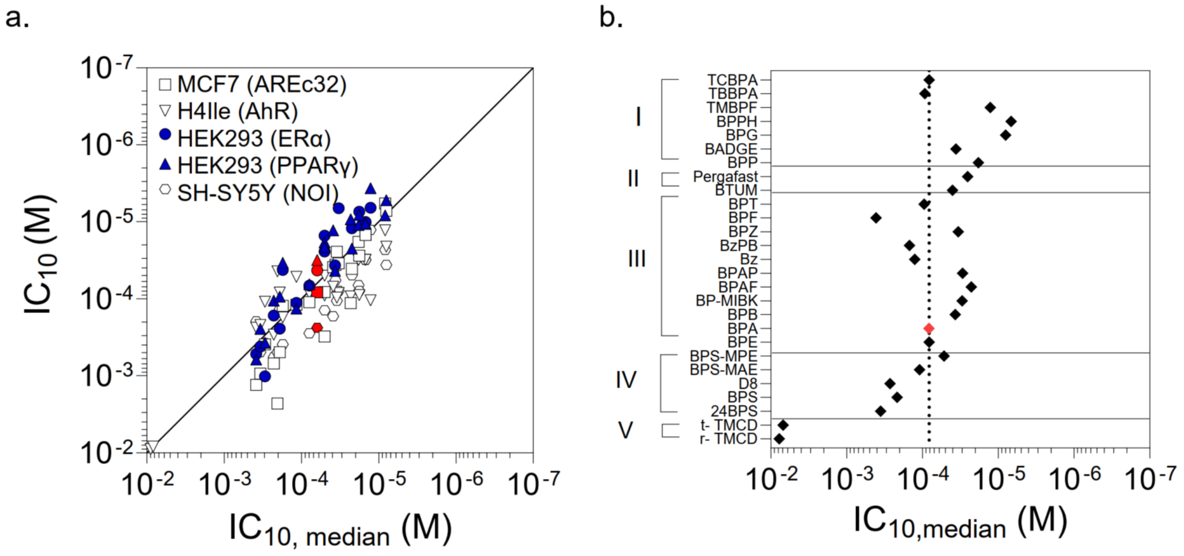

Figure S6: (a) Comparison of cytotoxicity IC_{10} for BPA and 26 alternatives across five different cell lines. The x-axis shows the median $IC_{10,median}$ (M), and the y-axis displays cytotoxicity IC_{10} (M) for individual cell lines on a logarithmic scale. (b) Median cytotoxicity ($IC_{10,median}$) of BPA and its alternatives grouped into five structural categories (I–V). The x-axis represents $IC_{10,median}$ (M) on a logarithmic scale, while the y-axis lists the tested bisphenols. Note that the axes are inverse because a lower IC_{10} relates to higher cytotoxicity. The dashed line indicates the IC_{10} value of BPA for comparison.

S5.2 Effect Concentrations and Specificity Ratio plots

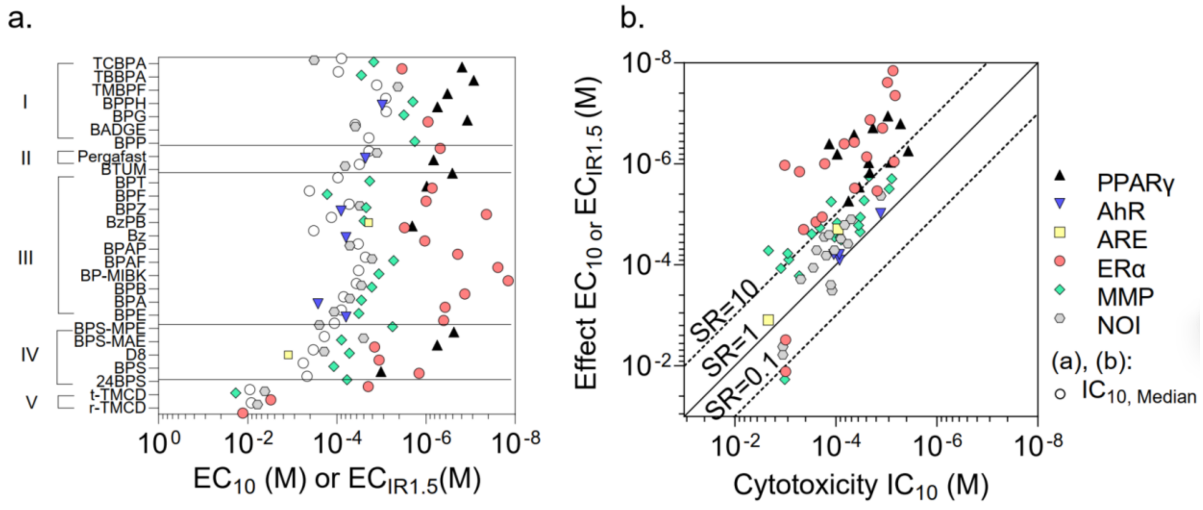

Figure S7: (a) Comparison of effect concentrations (EC_{10} or $EC_{IR1.5}$) and cytotoxicity $IC_{10,\ median}$ of 26 bisphenol alternatives and BPA for six specific endpoints. Specificity ratio ($SR_{cytotoxicity}$) analysis comparing experimental IC_{10} values (x-axis) with EC_{10} values (y-axis). The diagonal line represents $SR_{cytotoxicity}$=1, indicating no difference between effect (EC_{10}) and cytotoxicity (IC_{10}). Note that the axes are inverse because a lower IC_{10} relates to higher

cytotoxicity. Upward triangles represent PPARγ, downward triangles AhR, yellow squares ARE, red circle ERα, green diamond mitochondrial membrane potential (MMP) inhibition, grey hexagon neurite outgrowth inhibition (NOI) in differentiated SH-SY5Y, and white circle median cytotoxicity $IC_{10,median}$. Data in Table S6. BPA and its alternatives are categorized into different groups (I-V) based on their structural similarity.

S5.3: ERα- Efficacy

4,4'-Thiodiphenol (BPT) had the highest efficacy (80%), but Bisphenol S (BPS), Bisphenol F (BPF) and Bisphenol E (BPE) also demonstrated relatively high efficacy, with Emax% ranging between 50 and 60% (Figure S8a). All compounds are characterized by a free bridge atom (BPF, BPS, BPT) or only one methyl substituent (BPE), which influences the bridge and torsion angles between the phenolic rings[22] leading to more favorable stabilization of the active conformation of the ERα receptor (Figure S8b).[23]

a. b.

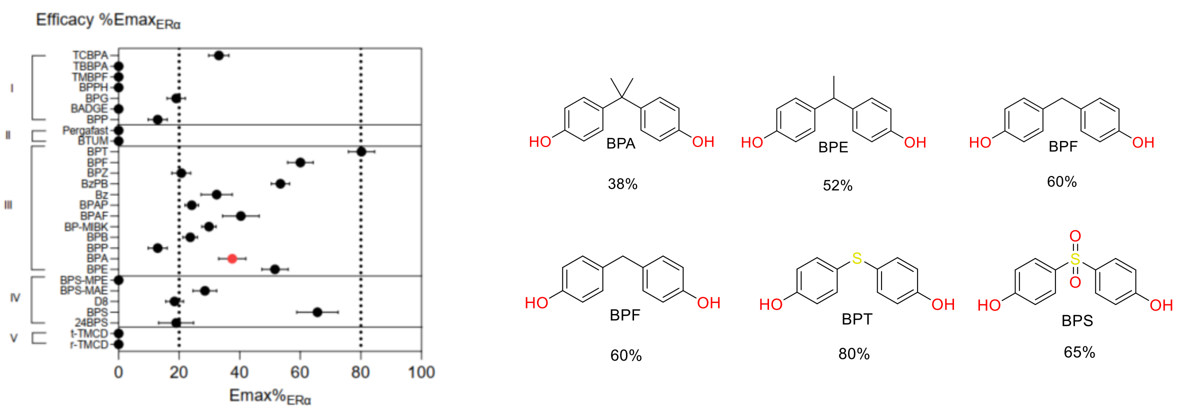

Figure S8: (a) Maximal Efficacy (Emax%) of 26 BPA alternatives in ERα-UAS-bla GripTite across different structural groups (I–V). The efficacy is expressed relative to E2. Dashed lines indicate the thresholds for partial agonism (20%) and full agonism (80%). Data points represent mean values with error bars indicating standard deviations (n=3). (b) Structure comparison of congeners with higher Emax%. Data in Table 1.

S5.4: Comparison with literature data for ERα reporter gene assays

To improve data comparability with our own results we converted reported EC_{50} to EC_{10} by dividing by 9, which is the ratio for log-logistic concentration-response curves with a hillslope of 1 (Figure S9a). Additionally, REP_{BPA} values were calculated to enable a more consistent evaluation. Methodological differences between studies must be considered, as Reiniger et al.[24] used the YES assay, while Goksøyr et al.[25] applied ERα from fish (Figure S9b).

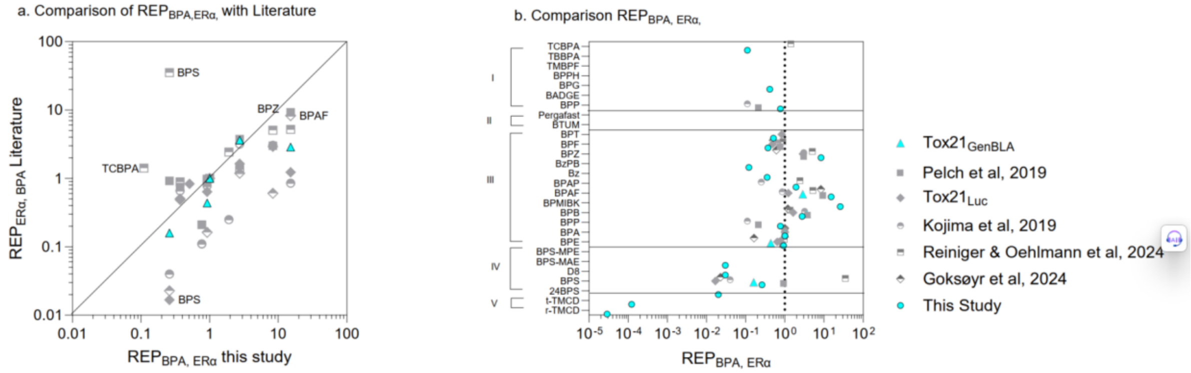

Figure S9: Comparison of Relative Effect Potency ($REP_{BPA,ER\alpha}$) between this study and literature data. (a) $REP_{BPA,ER\alpha}$ comparison, with the x-axis representing data from this study and the y-axis representing data reported in literature. The diagonal line indicates a 1:1 correlation, suggesting good agreement between both datasets. (b) Comparison of $REP_{BPA,ER\alpha}$ with literature data across different structural groups (I–V). Cyan symbols highlight the ER-UAS-bla GripTite (this study and $Tox21_{GenBLA}$).

S6: More details on the Cumulative Specificity Ratio Scoring

S6.1 Selection of the threshold and threshold function

The SR-Score concept builds on the concept of Specificity Ratios (SR),[15] which themselves are a derivative of Toxic Ratios (TR).[26, 27] Toxic Ratios compare observed cytotoxicity to predicted baseline toxicity ($IC_{10,\ baseline}$). A TR > 10 is commonly used as a threshold to indicate a specific mode of action, since at this level a clear distinction can be drawn between baseline toxicants and chemicals with specific effects. This threshold also reflects the uncertainty in the underlying mass balance model used to predict baseline toxicity.[27]

Analogous to the TR concept, a threshold value of 10 has also been proposed for SRs. While SRs between 1 and 10 are not considered irrelevant, they represent a zone of greater uncertainty with moderate specific effects. A classic stepwise threshold as usually applied with no transition zone is too strict to accommodate this uncertainty and could lead to uncertain conclusions.

Figure S10 illustrates three approaches for assigning the SR-Score based on the specificity ratio (SR): (a) a classical step-function threshold (eq. S19), (b) a linear ramp that increases from SR = x_0 to SR = 10 (eq. S20) and (c) a sigmoidal transformation (eq. S21) with adjustable center (x_0) and slope (k). At SR = 10, the assay is always considered "fully activated". The linear ramp function (magenta) assumes a direct proportional increase from SR = x_0 to SR = 10. The sigmoid function (k=1, x=5, orange) provides a smoother and flexible transition: It suppresses scores for low SR values, increases rapidly around SR ≈ x_0, and saturates at 1 for higher SR values. Its main advantage over the linear ramp is that its steepness can be adjusted independently from the starting-parameter x_0 and that it considers the contribution of weakly specifically acting compounds while they are fully excluded with the other threshold functions.

$$\text{SR-Score}_{\text{step}}=\begin{cases}0, & \text{SR}<x_0\\ 1, & \text{SR}\geq x_0\end{cases} \tag{S19}$$

$$\text{SR-Score}_{\text{linear}}=\begin{cases}0, & \text{SR}<x_0\\ \frac{\text{SR}}{10-x_0}, & x_0\leq\text{SR}\leq 10\\ 1, & \text{SR}\geq 10\end{cases} \tag{S20}$$

$$\text{SR-Score}_{\text{sigmoid}}=\frac{1}{1+e^{-(k(\text{SR}-x_0)}} \tag{S21}$$

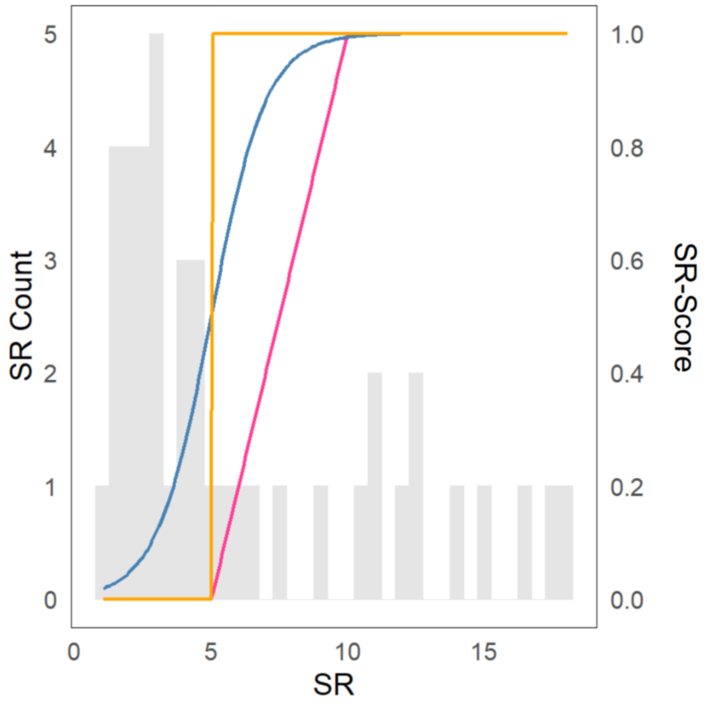

Figure S10: Comparison of stepwise function (eq. S19, orange), linear ramp-function (eq. S20, magenta), sigmoid function (eq. S21, blue) for deriving the SR-Score based on the specificity ratio SR. The histogram in the background (grey) depicts the SR distribution represented with a bin width of 0.5.

S6.2: Parameter Selection for the sigmoidal curve of the Σ-SR-Score

The center of the sigmoidal transformation was placed at an SR of 5 as a compromise that reflects the transition zone between baseline effects and clear specific effects. In contrast to applying a strict threshold at SR = 10, the sigmoidal transformation ensures that the score gradually increases towards 1 as SR approaches 10, while suppressing values near 1 to a score close to 0.

A sensitivity analysis of SR-Scores was performed by varying the k and x_0- parameters. Single-parameter changes of the sigmoid SR-score function were applied to simulated SRs ($1<SR<20$, $\Delta = 0.2$) and highlight the impact of slope (k) and midpoint (x_0) on score distribution (Figure S11). If k = 0.5, a very gradual curve includes nearly all low SR value with too many nonspecific results (Figure S11a). Increasing k from 0.5 to 4 sharpens the transition so strongly that scores are almost bimodally distributed, producing a nearly stepwise scoring function (Figure S11d). In practice, choosing k = 1 or 2 provides a good compromise (Figure S11b and c): it suppresses low SRs, fully includes values approaching SR = 10, and still allows moderate contributions in between. When comparing different center points, x_0 =5 positions the inflection point of the sigmoid SR-score function exactly at SR=5 allows the most symmetrical weighting of the score when SR=10 is set as an activity threshold.

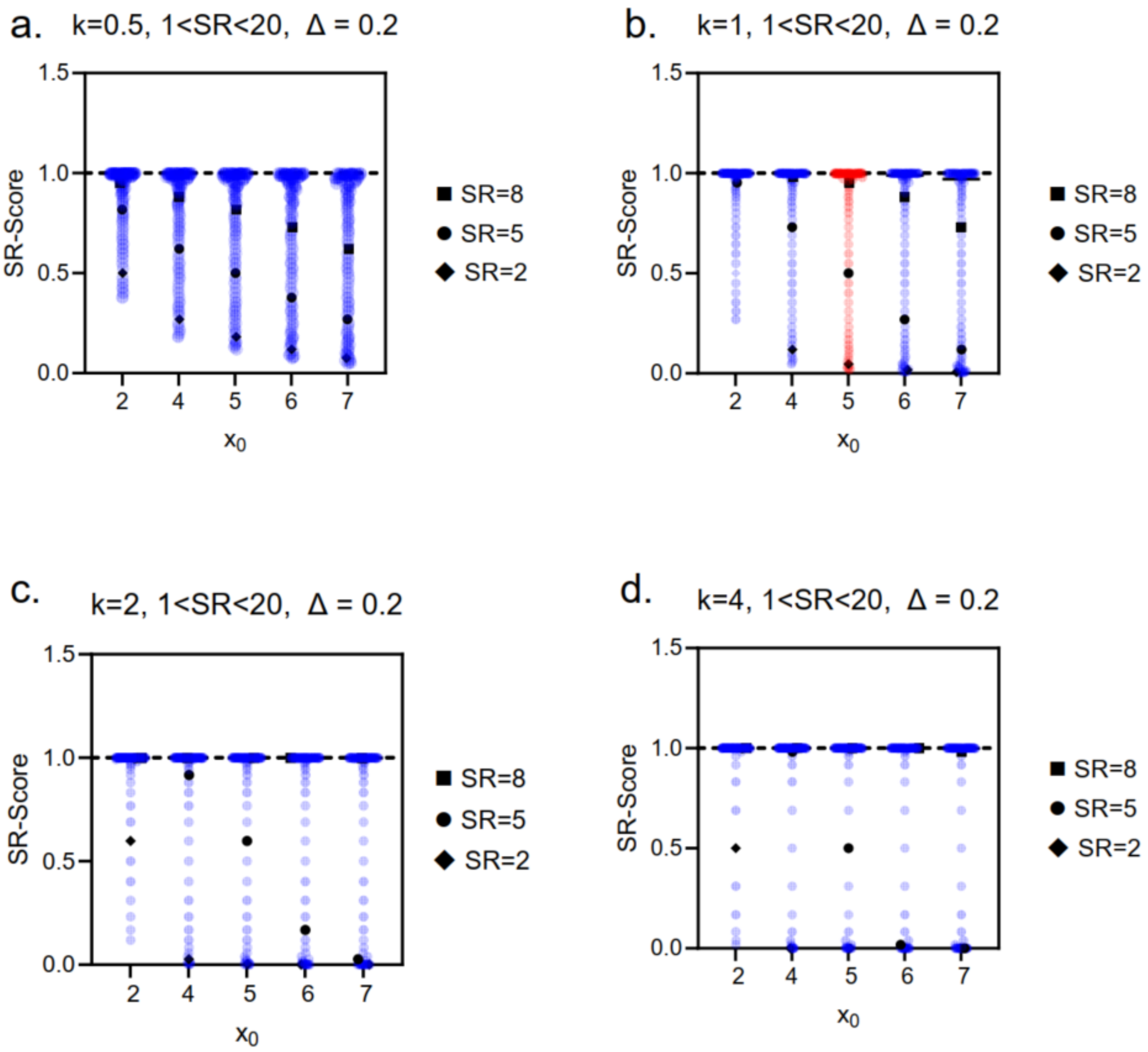

Figure S11: Sigmoid functions of simulated SRs (1<SR<20, step 0.2) with different parameters used for SR-Score calculation: (a) k= 0.5, (b) k= 1, (c) k = 2, (d) k= 4. The center parameter x_0 was varied between 2, 4, 5, 6 and 7.

S7. Comparison of Σ-SR-Score with ToxPi

ToxPi is another tool for integrating results across multiple *in vitro* assays. Similar to the Σ-SR-Score, ToxPi produces a dimensionless index score, calculated as a weighted combination of “slices” representing integrated data sources.[18, 28]

In their paper Reif et al.[18, 28] normalized effect potencies (e.g., $1/EC_{10}$) to the maximum value within each slice (e.g., assay) before summation (eq. S22).

$$\text{ToxPi-Score}=\sum_{n=1}^{i}\frac{\text{Assay}_i}{\max(\text{Assay}_i)}=\sum_{n=1}^{i}\frac{\log(\frac{1}{EC_{10,i}})}{\max(\log(\frac{1}{EC_{10,i}}))} \qquad \text{(S22)}$$

We applied the ToxPi methodology to our dataset and compared the resulting ToxPi ranks (Table S8) with those from the cumulative SR-scoring (Figure S12-13). The rankings agree for some substances (especially at the extremes) but differ for many others.

An advantage and novelty of the cumulative SR-Score is that it reflects how many assays in a test battery show specific activation. Thus, substances with a broad activity profile rank highly. As a result, the instance of an individual high potency substance disproportionately influencing the ranking is much reduced. In contrast, the ToxPi score is strongly driven by the potency observed in individual assays, without accounting for cytotoxicity burst artefacts. For example, BPZ achieves a relatively high ToxPi Score due to its strong ERα activity but only a SR-Score of 1, whereas 2,4-BPS, which shows relevant activation in both ERα and MMP assays, ranks much lower with ToxPi due to the low effect potencies. The normalization of slices to their maxima (most potent assay result) also leads to a high sensitivity of ToxPi-Scores to data gaps. This is not the case for the Cumulative SR-Score.

One major disadvantage of the Σ-SR-Score is that it is strongly dependent on the availability of matched cytotoxicity data (IC_{10}). If no experimental cytotoxicity data are available, they have to be substituted by $IC_{10,median}$ of other cell lines or be derived from predicted $IC_{10,baseline}$ values. Another limitation is that it neglects the variability of different assays e.g., sensitivity and dynamic range, which we have not yet adjusted for during our sigmoidal transformation step, and which potentially introduces biases. Then, as mentioned above, the interpretability becomes more difficult for larger numbers of assays and the choice of the threshold (center of sigmoidal curve) is crucial and has to be decided carefully.

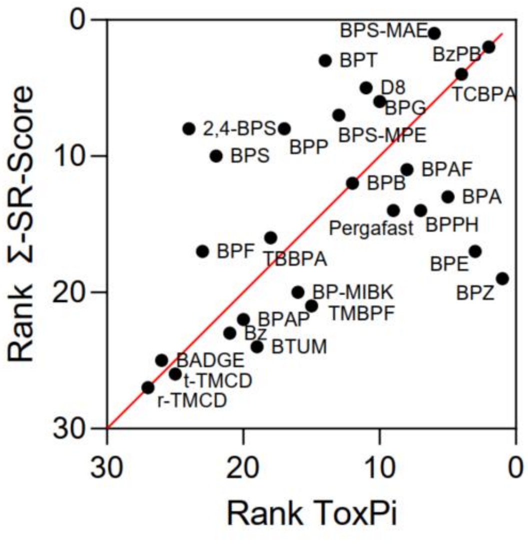

Figure S12: Plot comparing the ranking of both scoring approaches, ToxPi on the x-axis, and Cumulative SR-Score (Σ-SR-Score) on the y-axis.

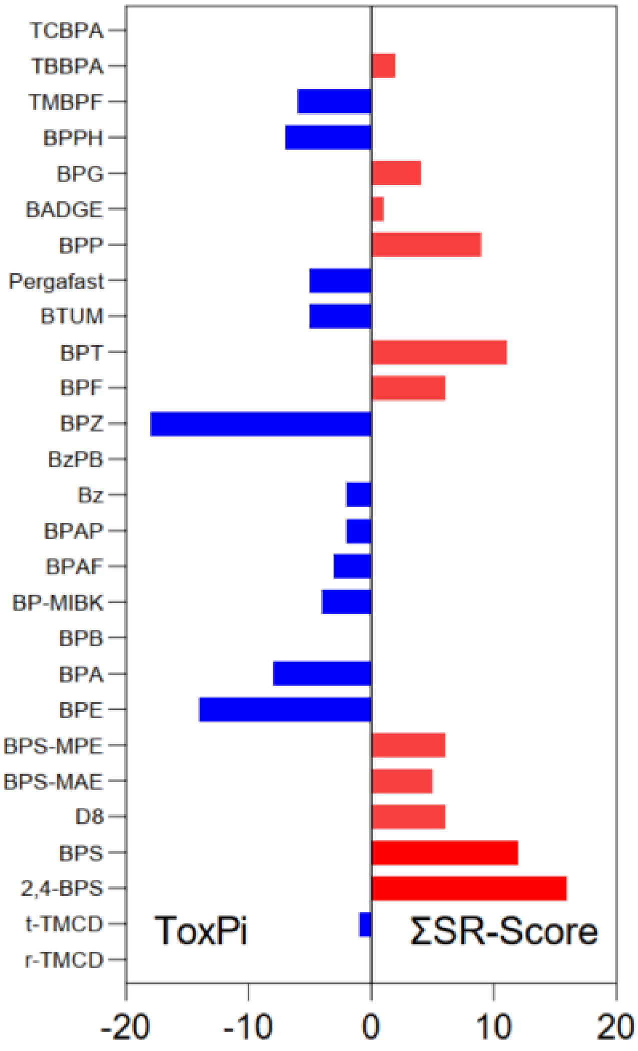

Figure S13: Comparing the differences in rankings between ToxPi -Score and Cumulative SR-Score (Σ-SR-Score).

S8: Abiotic CYP combined with bioassay testing

S8.1. Identification of the oxidation product of BPT with high-resolution mass spectrometry.

The chromatogram in Figure S14 displays the retention time distribution of detected compounds in the BPT abiotic cytochrome enzyme aCyp mix after 15 min incubation, with a clear peak at 9.99 min, indicating the presence of an oxidation product. The MS spectrum confirms the identity of this product by the high-resolution m/z 255.0426 peak, suggesting a single hydroxylation event.

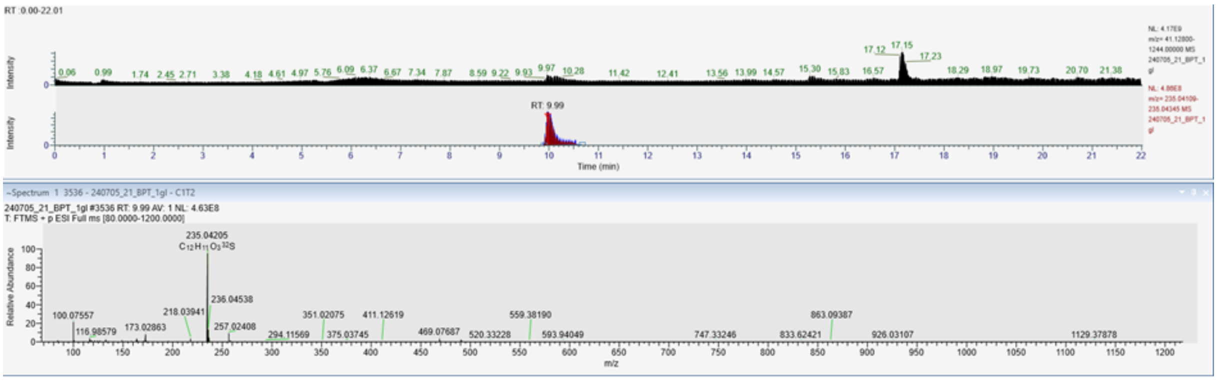

Figure S14: LC-HRMS chromatogram and mass spectrum of BPT after aCYP oxidation, showing the extracted ion chromatogram (EIC) with a retention time of 9.99 min. The corresponding high-resolution mass spectrum identifies a mono-hydroxylated oxidation product, with the detected ion at m/z 255.0426, corresponding to the molecular formula $C_{13}H_{10}NO_4S$.

S8.2. TK-Ratio combined with C_{parent} ratio analysis.

While C_{parent} measurements in Table S9 were performed independently from the bioassays that were used to calculate the TK-Ratio (Table S10), we also performed a combined experiment, where C_{parent} was measured alongside the bioassay (Table S11). Overall, the experiments agreed well, but the comparison also shows the large variability of the C_{parent}-Ratios in both experiments especially pronounced in the combined experiment (Figure S15a). However, the estrogenicity was remarkable similar in both experiments (Figure S15b).

For several BPA alternatives, the observed cytotoxicity was lower than expected from measured C_{parent} concentration (e.g., the hydrophobic chemicals in Group I, 2,2-Bis(2-hydroxy-5-biphenylyl)propane (BPPH), BPG, or 4-((4-Isopropoxyphenyl)sulfonyl)phenol (D8), Bisphenol B (BPB).

Among those, the very hydrophobic molecules were not oxidized to such an extent to explain the loss of toxicity. They were most likely not dissolved in the assay medium and stuck to the film of the catalyst that forms during the blowdown step. This might explain why we observed lower TK-Ratios for cytotoxicity than expected from the C_{parent} data.

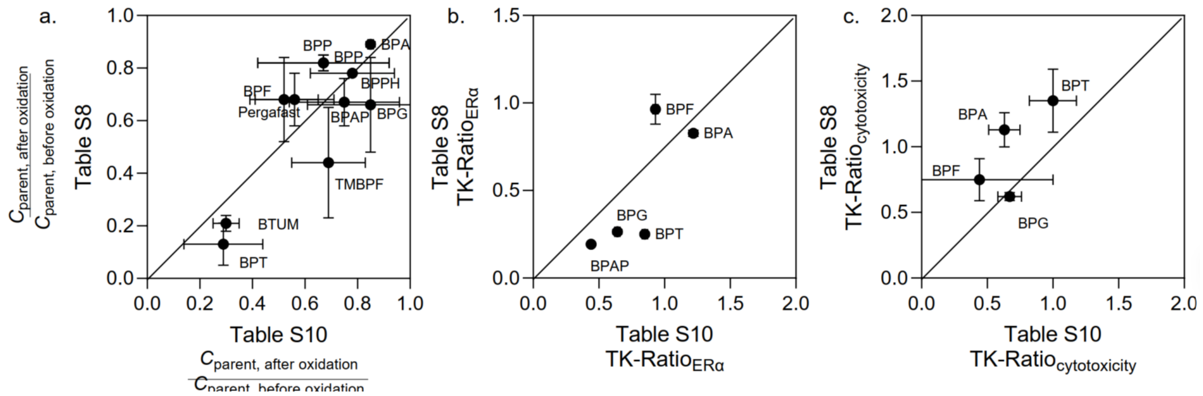

Figure S15: (a) Remaining parent compound (C_{parent}-Ratio) after abiotic CYP oxidation, (b) TK-Ratio of ERα activity EC_{10} and (c) cytotoxicity (IC_{10}). Data from Tables S9 to S11.

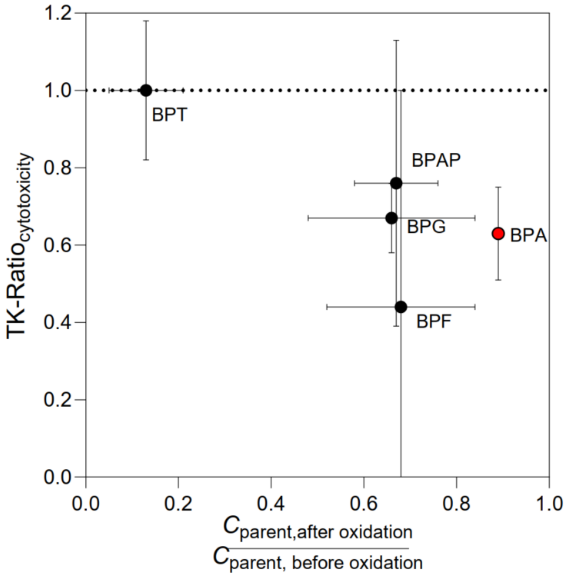

Figure S16:TK-ratio vs c_{parent}-ratio. TK-Ratio = 1 – no change in toxicity, TK-Ratio > 1 increased toxicity, TK-Ratio < 1 detoxification. Data from Table S11.

References

(1) Niu, L.; Henneberger, L.; Huchthausen, J.; Krauss, M.; Ogefere, A.; Escher, B. I. pH-Dependent Partitioning of Ionizable Organic Chemicals between the Silicone Polymer Polydimethylsiloxane (PDMS) and Water. *ACS Environ Au* **2022**, *2* (3), 253–262. DOI: 10.1021/acsenvironau.1c00056

(2) Isik, M.; Levorse, D.; Mobley, D. L.; Rhodes, T.; Chodera, J. D. Octanol-water partition coefficient measurements for the SAMPL6 blind prediction challenge. *J Comput Aided Mol Des* **2020**, *34* (4), 405–420. DOI: 10.1007/s10822-019-00271-3

(3) Avdeef, A. pH-Metric log P. Part 1. Difference Plots for Determining Ion-Pair Octanol-Water Partition Coefficients of Multiprotic Substances. *Quantitative Structure-Activity Relationships* **2008**, *11* (4), 510–517. DOI: 10.1002/qsar.2660110408

(4) Escher, B. I.; Schwarzenbach, R. P. Partitioning of Substituted Phenols in Liposome−Water, Biomembrane−Water, and Octanol−Water Systems. *Environ Sci Technol* **1996**, *30* (1), 260–270. DOI: 10.1021/es9503084

(5) Endo, S.; Escher, B. I.; Goss, K. U. Capacities of membrane lipids to accumulate neutral organic chemicals. *Environ Sci Technol* **2011**, *45* (14), 5912–5921. DOI: 10.1021/es200855w

(6) Qin, W.; Henneberger, L.; Gluge, J.; Konig, M.; Escher, B. I. Baseline Toxicity Model to Identify the Specific and Nonspecific Effects of Per- and Polyfluoroalkyl Substances in Cell-Based Bioassays. *Environ Sci Technol* **2024**, *58* (13), 5727–5738. DOI: 10.1021/acs.est.3c09950

(7) Henneberger, L.; Goss, K. U.; Endo, S. Equilibrium Sorption of Structurally Diverse Organic Ions to Bovine Serum Albumin. *Environ Sci Technol* **2016**, *50* (10), 5119–5126. DOI: 10.1021/acs.est.5b06176

(8) Endo, S.; Bauerfeind, J.; Goss, K. U. Partitioning of neutral organic compounds to structural proteins. *Environ Sci Technol* **2012**, *46* (22), 12697–12703. DOI: 10.1021/es303379y

(9) Huchthausen, J.; Braasch, J.; Escher, B. I.; Konig, M.; Henneberger, L. Effects of Chemicals in Reporter Gene Bioassays with Different Metabolic Activities Compared to Baseline Toxicity. *Chem Res Toxicol* **2024**, *37* (5), 744–756. DOI: 10.1021/acs.chemrestox.4c00017

(10) Wang, D.; Zhao, H.; Fei, X.; Synder, S. A.; Fang, M.; Liu, M. A comprehensive review on the analytical method, occurrence, transformation and toxicity of a reactive pollutant: BADGE. *Environ Int* **2021**, *155*, 106701. DOI: 10.1016/j.envint.2021.106701

(11) Eckardt, M.; Simat, T. J. Bisphenol A and alternatives in thermal paper receipts - a German market analysis from 2015 to 2017. *Chemosphere* **2017**, *186*, 1016–1025. DOI: 10.1016/j.chemosphere.2017.08.037

(12) European Chemical Agency (ECHA). *2,2,4,4-tetramethylcyclobutane-1,3-diol, mixed isomers - ECHA Chemical Database*. 2025. https://chem.echa.europa.eu/100.019.219/dossier-view/e47a2ef2-3848-4538-9a24-16445e0731f5/IUC5-7ec5ad9b-bd2c-4973-9a76-ac87f2b914fd_00dbfa3d-a0f4-41b2-93f3-1d22f25ae08e?searchText=TMCD (accessed 2025/07/04).

(13) Konig, M.; Escher, B. I.; Neale, P. A.; Krauss, M.; Hilscherova, K.; Novak, J.; Teodorovic, I.; Schulze, T.; Seidensticker, S.; Kamal Hashmi, M. A.; Ahlheim, J.; Brack, W. Impact of untreated wastewater on a major European river evaluated with a combination of in vitro bioassays and chemical analysis. *Environ Pollut* **2017**, *220* (Pt B), 1220–1230. DOI: 10.1016/j.envpol.2016.11.011

(14) Lee, J.; Escher, B. I.; Scholz, S.; Schlichting, R. Inhibition of neurite outgrowth and enhanced effects compared to baseline toxicity in SH-SY5Y cells. *Arch Toxicol* **2022**, *96* (4), 1039–1053. DOI: 10.1007/s00204-022-03237-x

(15) Escher, B. I.; Henneberger, L.; Konig, M.; Schlichting, R.; Fischer, F. C. Cytotoxicity Burst? Differentiating Specific from Nonspecific Effects in Tox21 in Vitro Reporter Gene Assays. *Environ Health Perspect* **2020**, *128* (7), 77007. DOI: 10.1289/EHP6664

(16) Escher, B. I.; Neale, P. A.; Villeneuve, D. L. The advantages of linear concentration-response curves for in vitro bioassays with environmental samples. *Environ Toxicol Chem* **2018**, *37* (9), 2273–2280. DOI: 10.1002/etc.4178

(17) Feshuk, M.; Kolaczkowski, L.; Dunham, K.; Davidson-Fritz, S. E.; Carstens, K. E.; Brown, J.; Judson, R. S.; Paul Friedman, K. The ToxCast pipeline: updates to curve-fitting approaches and database structure. *Front Toxicol* **2023**, *5*, 1275980. DOI: 10.3389/ftox.2023.1275980

(18) Filer, D.; Patisaul, H. B.; Schug, T.; Reif, D.; Thayer, K. Test driving ToxCast: endocrine profiling for 1858 chemicals included in phase II. *Curr Opin Pharmacol* **2014**, *19*, 145–152. DOI: 10.1016/j.coph.2014.09.021

(19) Braun, G. AutomatedBioassayScreening, https://git.ufz.de/braung/automatedbioassayscreening [accessed 2025/07/19]. **2023**.

(20) Braun, G.; Herberth, G.; Krauss, M.; König, M.; Wojtysiak, N.; Zenclussen, A. C.; Escher, B. I. Neurotoxic mixture effects of chemicals extracted from blood of pregnant women. *Science* **2024**, *386* (6719), 301–309. DOI: 10.1126/science.adq0336

(21) Braun, G.; Krauss, M.; Escher, B. I. Recovery of 400 Chemicals with Three Extraction Methods for Low Volumes of Human Plasma Quantified by Instrumental Analysis and In Vitro Bioassays. *Environ Sci Technol* **2023**, *57* (48), 19363–19373. DOI: 10.1021/acs.est.3c05962

(22) Lim, C. F.; Tanski, J. M. Structural Analysis of Bisphenol-A and its Methylene, Sulfur, and Oxygen Bridged Bisphenol Analogs. *Journal of Chemical Crystallography* **2007**, *37* (9), 587–595. DOI: 10.1007/s10870-007-9207-8

(23) Delfosse, V.; Grimaldi, M.; Pons, J. L.; Boulahtouf, A.; le Maire, A.; Cavailles, V.; Labesse, G.; Bourguet, W.; Balaguer, P. Structural and mechanistic insights into bisphenols action provide guidelines for risk assessment and discovery of bisphenol A substitutes. *Proc Natl Acad Sci U S A* **2012**, *109* (37), 14930–14935. DOI: 10.1073/pnas.1203574109
(24) Reininger, N.; Oehlmann, J. Regrettable substitution? Comparative study of the effect profile of bisphenol A and eleven analogues in an in vitro test battery. *Environ Sci Europe* **2024**, *36*. DOI: 10.1186/s12302-024-00900-1
(25) Goksøyr, S. Ø.; Yadetie, F.; Johansen, C. T.; Jacobsen, R. G.; Lille-Langøy, R.; Goksøyr, A.; Karlsen, O. A. Interaction of Bisphenol A and Its Analogs with Estrogen and Androgen Receptor from Atlantic Cod (Gadus morhua). *Environ Sci Technol* **2024**, *58* (32), 14098–14109. DOI: 10.1021/acs.est.4c01500
(26) Verhaar, H. J. M.; Van Leeuwen, C. J.; Hermens, J. L. M. Classifying environmenta pollutants .1. Structure-activity-relationships for prediction of aquatic toxicity. *Chemosphere* **1992**, *25* (4), 471–491. DOI: 10.1016/0045-6535(92)90280-5
(27) Maeder, V.; Escher, B. I.; Scheringer, M.; Hungerbühler, K. Toxic Ratio as an Indicator of the Intrinsic Toxicity in the Assessment of Persistent, Bioaccumulative, and Toxic Chemicals. *Environ. Sci. Technol.* **2004**, *38* (13), 3659–3666. DOI: 10.1021/es0351591
(28) Reif, D. M.; Martin, M. T.; Tan, S. W.; Houck, K. A.; Judson, R. S.; Richard, A. M.; Knudsen, T. B.; Dix, D. J.; Kavlock, R. J. Endocrine profiling and prioritization of environmental chemicals using ToxCast data. *Environ Health Perspect* **2010**, *118* (12), 1714–1720. DOI: 10.1289/ehp.1002180
